# Autonomic Effects of Music in Health and Crohn's Disease: The Impact of Isochronicity, Emotional Valence, and Tempo

**DOI:** 10.1371/journal.pone.0126224

**Published:** 2015-05-08

**Authors:** Roland Uwe Krabs, Ronny Enk, Niels Teich, Stefan Koelsch

**Affiliations:** 1 Max Planck Institute for Human Cognitive and Brain Sciences, Leipzig, Germany; 2 Group practice for Digestive and Metabolic Diseases, Leipzig, Germany; 3 Cluster of Excellence “Languages of Emotions”, Freie Universität Berlin, Berlin, Germany; University Zurich, SWITZERLAND

## Abstract

**Background:**

Music can evoke strong emotions and thus elicit significant autonomic nervous system (ANS) responses. However, previous studies investigating music-evoked ANS effects produced inconsistent results. In particular, it is not clear (a) whether simply a musical tactus (without common emotional components of music) is sufficient to elicit ANS effects; (b) whether changes in the tempo of a musical piece contribute to the ANS effects; (c) whether emotional valence of music influences ANS effects; and (d) whether music-elicited ANS effects are comparable in healthy subjects and patients with Crohn´s disease (CD, an inflammatory bowel disease suspected to be associated with autonomic dysfunction).

**Methods:**

To address these issues, three experiments were conducted, with a total of *n* = 138 healthy subjects and *n* = 19 CD patients. Heart rate (HR), heart rate variability (HRV), and electrodermal activity (EDA) were recorded while participants listened to joyful pleasant music, isochronous tones, and unpleasant control stimuli.

**Results:**

Compared to silence, both pleasant music and unpleasant control stimuli elicited an increase in HR and a decrease in a variety of HRV parameters. Surprisingly, similar ANS effects were elicited by isochronous tones (i.e., simply by a tactus). ANS effects did not differ between pleasant and unpleasant stimuli, and different tempi of the music did not entrain ANS activity. Finally, music-evoked ANS effects did not differ between healthy individuals and CD patients.

**Conclusions:**

The isochronous pulse of music (i.e., the tactus) is a major factor of music-evoked ANS effects. These ANS effects are characterized by increased sympathetic activity. The emotional valence of a musical piece contributes surprisingly little to the ANS activity changes evoked by that piece.

## Introduction

Emotions and their peripheral-physiological components are subjects of intensive research [1]. The activities of the autonomic nervous system (ANS) that accompany emotions are studied with especially great interest due to the ANS′s relevance for a broad range of pathologies, such as cardiovascular diseases [2,3], chronic diseases of the immune system [4,5,6,7], and affective disorders [8]. The ANS has two branches: the sympathetic nervous system and the parasympathetic nervous system. Their centers in the brainstem (such as the parabrachial nucleus) and the hypothalamus are influenced by numerous brain structures implicated in emotion, such as the amygdala, insula, orbitofrontal cortex, cingulate cortex, hippocampus, and ventral tegmental area [9,10]. As a result, emotional processes can impact via the ANS on all peripheral organ systems [11].

Music has the power to evoke strong emotions [12]. Accordingly, functional neuroimaging studies have shown that music-evoked emotions can change activity in virtually all structures of the Greater Limbic System [9], including those involved in the modulation of ANS activity mentioned above [13]. Thus, music is capable of modulating ANS activity. However, as reviewed further below, there is a scarcity of systematic research on how musical parameters (such as tempo or consonance/dissonance) influence ANS activity as indexed by heart rate, heart rate variability, and electrodermal activity.

### Music and ANS activity

In the following sections, we will review studies investigating effects of music on ANS activity. For this purpose, we searched the PubMed/MEDLINE database (using the search terms “music AND heart rate”, “music AND heart rate variability”, “music AND electrodermal”, as well as “music AND skin”) for studies that were performed with healthy participants in the absence of physical or psychological stressors. Thus, only studies were included in which ANS reactions to music would not be biased by pathological conditions or stress-evoked shifts in ANS balance. We excluded studies reporting data only after, but not during, music listening [14,15,16,17,18,19,20]. Furthermore, studies using stimuli shorter than 30 seconds [21,22] were excluded due to potential bias caused by orienting and startle responses. Moreover, in order to compare the results of ANS parameters during music listening to a reference value, we only included studies that reported silence or other non-musical baseline measurements (which were made, e.g., during listening to instructions or filling out questionnaires).

### Heart rate (HR)

HR is the frequency at which the heart beats. It is regulated by numerous reflex-like circuits involving both subcortical brain structures and intra-thoracic cardiac ganglia [23], which are in turn influenced by the amygdala and cortical forebrain structures involved in emotion [10]. Generally, emotional arousal is associated with sympathetic ANS activity [1], thus leading to an increase in HR, whereas parasympathetic ANS activity leads to a decrease in HR [24]. Accordingly, several studies reported (trends towards) higher HR with arousing music compared to tranquilizing or less arousing music [25,26,27,28,29,30,31,32,33]. However, when comparing HR during music listening to baseline measurements, previous studies do not provide a consistent picture: Although some studies demonstrated an increase in HR with arousing music and a decrease in HR with tranquilizing music [25,27], others reported increases in HR with arousing as well as tranquilizing music [26,34], decreases in HR with tranquilizing as well as arousing music [29,35], or differences in HR despite similar music-evoked arousal [36]. Therefore, factors other than arousal also play a role in music-evoked HR effects. One factor might be music-evoked emotional valence, and several studies reported that, compared to negative valence (displeasure), positive valence (pleasure) is associated with higher HR [28,29,36]. However, in two of these studies [28,29], emotional valence confounded with emotional arousal (e.g., happy stimulating music was compared to sad tranquilizing music), making it difficult to clarify effects with respect to music-evoked emotional valence. Moreover, other studies did not demonstrate effects of music-evoked emotional valence on HR [35,37,38]. Thus, the impact of emotional valence on HR has remained elusive.

Surprisingly, despite the well-described neural connections between music perception, emotion, and the ANS (as outlined above), many studies found no significant HR effects with music listening compared to baseline values [39,40,41,42,43,44,45]. A lack of statistical power may explain the missing effects because some of these studies had small numbers of participants (e.g., 18 [39,44]), or performed only few trials of music listening, thus increasing the risk of a low signal-to-noise ratio (e.g., three trials in [40,44,45] and only one trial in [41]). Generally, the methods of previous studies on music-evoked HR effects were very heterogeneous: For example, some authors let the participants choose the music [28,39], others presented instrumental tunes as well as music with vocals [25,33,42]. In summary, it is unclear how HR is influenced by (a) listening to music compared to resting in silence, and (b) by the emotional valence evoked by the music.

### Heart rate variability (HRV)

HRV describes the variability of the HR interbeat intervals. From these intervals, several time domain, frequency domain, and nonlinear HRV parameters can be obtained that are associated with sympathetic or parasympathetic modulation (for details, see Table 1 and [46,47]). Thus, HRV has become an established indicator of ANS activity and sympathetic/parasympathetic (i.e., sympathovagal) balance [46]. However, there is debate regarding the accuracy of the autonomic associations of single HRV parameters [48]. Similar to HR, HRV is modulated by limbic and paralimbic brain structures and is thus affected by emotional processes [49]. Only a few studies have investigated the effects of music listening on HRV [50], and most were conducted in clinical settings (for reviews see [51,52,53]). As for HR, emotional arousal is discussed as a major factor for effects of music on HRV and results from several studies imply that different levels of subjective arousal during music listening determine different HRV [25,26,34,54,55,56]. However, other studies did not demonstrate clear relationships of arousal and HRV (despite demonstrating significant HRV effects during music listening compared to baseline values [29,30,45,57]). This indicates that additional factors impact on music-evoked HRV effects. Data on music-evoked emotional valence and HRV is scarce and did not demonstrate different HRV with different levels of pleasure or displeasure [29,37]. Moreover, as for HR, several studies did not report any HRV effects of listening to music compared to baseline values [41,58,59], adding to the inconsistent picture concerning music and HRV. As with studies on music and HR, the methods across studies on music and HRV were very heterogeneous (e.g., only a single piece of music was presented [30,41] or a song with vocals was compared to an instrumental tune [54,55,56,57,59]). Moreover, because most of the cited studies on music and HRV were performed with small numbers of participants (e.g., 10 [58] or 12 [55,56]), minor effects of music listening on HRV may not have been detected. Therefore, as for HR, it is not clear how HRV is influenced by (a) music listening compared to resting in silence, and (b) by the emotional valence evoked by the music.

**Table 1:** Parameters of heart rate variability (HRV).

	Parameter	Description	Comment
Time domain parameters	SDNN	Standard deviation of all normal-to-normal interbeat intervals	Estimate of overall HRV
	RMSSD	Square root of mean sum of squares of successive interbeat differences	Estimate of short term components of HRV
Frequency domain parameters	HF	Power of high frequency range (.15 - .4 Hz)	Corresponds to respiratory sinus arrhythmia, associated with parasympathetic activity
	HF n.u.	HF power in normalized units (HF / (total power—very low frequency power) x 100)	
	LF	Power of low frequency range (.04 - .15 Hz)	Corresponds to barocepter reflexes, autonomic association inconclusive
	LF n.u.	LF power in normalized units (LF / (total power—very low frequency power) x 100)	
	LF/HF	Ratio LF / HF	
Nonlinear parameters	SD1	Standard deviation of width of Poincaré plot (current interbeat interval length plotted against preceding)	Estimate of short term HRV
	SD2	Standard deviation of length of Poincaré plot (current interbeat interval length plotted against preceding)	Estimate of total HRV

### Electrodermal activity (EDA)

EDA is a measure of activity of the eccrine sweat glands located on the palms of hands and feet. These glands are innervated by sympathetic fibers. As for HR and HRV, limbic and paralimbic brain structures modulate the autonomic centers underlying EDA [60]. Thus, emotional processes can elicit sweating on the palms of hands and feet, independently of the thermoregulation of the body [60]. The effects of music on EDA were reported only in few non-clinical studies [28,29,33,35,41,43,44]. As for HR, it has been commonly reported that higher music-evoked emotional arousal is associated with greater EDA compared to lower emotional arousal [28,29,35,44], even though some studies did not demonstrate different EDA with different levels of arousal [33,43]. However, again as for HR, the data of EDA effects during music listening compared to baseline values is conflicting: Several studies demonstrated increases in EDA with arousing music [28,35,44] or tranquilizing music [41], whereas others reported decreases in EDA with arousing music [29,33,43] or tranquilizing music [29,43]. Therefore, as for HR and HRV, additional factors besides arousal play a role in music-evoked EDA effects. Concerning music-evoked emotional valence, some studies reported that positive valence is associated with higher EDA compared to negative valence [28,29,35]. However, in all of these studies, music-evoked emotional valence confounded with music-evoked emotional arousal. Moreover, other studies did not demonstrate effects of music-evoked emotional valence on EDA [38,61]. Thus, the impact of this factor on EDA is not clear. Similar methodological issues concerning the musical stimuli and numbers of participants exist with these studies as for studies on HR and HRV. In summary, it is unclear how EDA is influenced by emotional valence during music listening compared to resting in silence.

### Experimental rationale

As described above, the evidence regarding the ANS effects of music is inconclusive, often even contradictory, and many authors in this field called for additional studies [15,17,32,45,56]. This situation is surprising because psychological models on music and emotion consistently include ANS activity changes as a core factor contributing to music-evoked emotion [62,63,64]. Several theoretical accounts have even assumed that the entrainment of ANS processes with music plays a role for music-evoked emotion [62,63,65]. The contradictory findings on ANS effects of music are also surprising given the established potential of music to reduce stress, pain, and anxiety via neuroimmunological as well as neuroendocrine effects [65,66,67], in which the ANS is likely to play a pivotal role. As elaborated on in the previous sections, music-evoked emotional arousal is the factor reported most consistently to influence the ANS effects of music. However, arousal does not conclusively explain the inconsistent ANS effects of music listening compared to baseline values. Therefore, we presented a variety of joyful pleasant music pieces to several groups of participants to establish the ANS effects reliably elicited by these pieces compared to resting in silence. Furthermore, we investigated several factors likely to impact on the ANS besides emotional arousal during music listening.

**Fig 1:**
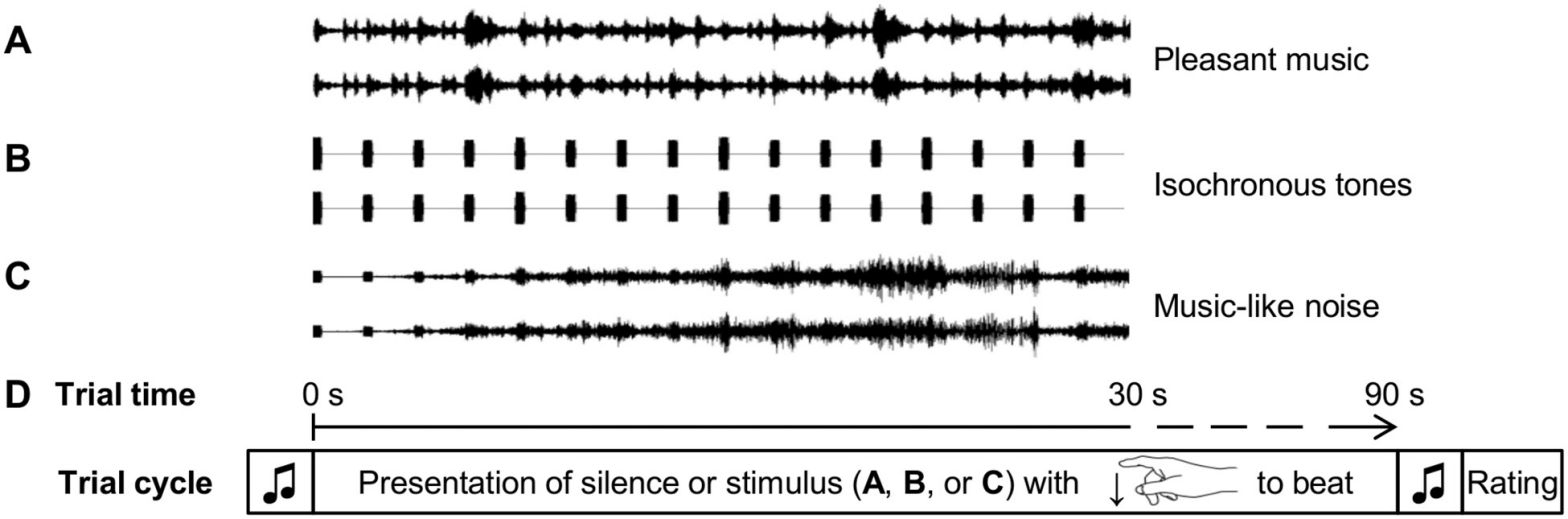
Stimuli and procedure of Experiment 1. A, B, and C show 30 second samples of a triplet of corresponding acoustic stimuli used. Notice that the isochronous tones in B and C have identical tempi as the stimulus in A. D demonstrates the sequence of one trial. Twenty-five such trials with different stimuli were presented. Before and after each auditory stimulus, short sinus tone sequences (indicated by the two notes in the figure) were used to notify the subjects that a trial will start, respectively the rating procedure will follow.

Firstly, the role of the tactus of music was tested. This has never been studied systematically before. Most music (except, e.g., some meditation music or so-called new age music) is based on an isochronous pulse. This pulse is commonly perceived as the tactus or “beat” of the music (see Fig 1). Whether such a tactus alone has an effect on ANS activity has remained unclear. Secondly, whether the tempo of this tactus or the quality of emotional valence evoked by music listening (pleasure or displeasure) have systematic ANS effects is not clear. Therefore, we designed a series of three experiments to test (a) whether merely a tactus is sufficient to elicit reliable effects on ANS activity as measured by HR, HRV, and EDA; (b) whether the tempo of the music impacts on the ANS activity (for details see Experiment 2) and (c) whether music-evoked emotional valence modulates these effects. Finally, to explore the diagnostic and therapeutic utility of joyful music (as used in this study), we tested whether ANS reactions to music differ between patients with Crohn´s disease (CD) and healthy subjects. CD patients were chosen because disease-related dysfunctions of the ANS have been reported with CD, and further data of the ANS activity is needed in this group of patients (for details see Experiment 3).

## Experiment 1: The ANS response to music, isochronous tones, and music-like noise

As reviewed above, the effects of music on HR, HRV, and EDA reported in previous studies were not consistent. Furthermore, it is not known whether “real music”, i.e., encompassing a tactus, rhythm, melody, and harmony, and thus expressing emotions, is required to elicit ANS responses, or whether simply a musical tactus (i.e., an isochronous auditory pulse) is sufficient. Moreover, it is unclear whether positive or negative valence evoked by a music stimulus influences such responses. To investigate these issues, we presented healthy subjects with (a) pleasant joyful music, (b) isochronous complex tones with only minimal melodic information, and (c) isochronous complex tones overlaid with unpleasant dissonant music-like noise (for an illustration see Fig 1). Importantly, all stimuli had identical tempo (i.e., identical numbers of beats per minute, bpm). In addition, we employed silence trials without any stimulus presentation. The comparison between pleasant music and silence tested the hypothesis that pleasant music would evoke significant changes in HR, HRV, and EDA. Due to the inconsistent results of previous studies, no directed hypotheses were made. Moreover, the comparison between isochronous tones (which were expected to have neutral valence) and silence tested whether simply isochronous tones are able to elicit ANS responses. Lastly, the comparison between pleasant and unpleasant stimuli tested whether responses in HR, HRV, and EDA are influenced by the music-evoked emotional valence.

### Methods

#### Ethics statement

The study was approved by the ethics committee of the University of Leipzig. Written informed consent was obtained from all participants.

#### Subjects

Data were obtained from 76 participants (36 female, aged 18 to 35 years). They were randomly selected from an institutional database of healthy volunteers of the Max Planck Institute for Human Cognitive and Brain Sciences (health was assessed by interviews before acceptance into the database). Exclusion criteria for this study comprised any history of somatic or psychiatric disease (including hearing disorders and diseases interfering with ANS activity such as diabetes mellitus or affective disorders). To reassure subject´s health, they were interviewed again on the day of the experiment.

#### Stimuli

Three types of acoustical stimuli were used (see Fig 1): (a) pleasant joyful music, (b) isochronous Shepard tones, and (c) isochronous Shepard tones overlaid with unpleasant music-like noise. (a) *Pleasant music* stimuli were excerpts of joyful instrumental tunes from a variety of epochs and genres (encompassing classical, jazz, and folk music). Thus, the influence of individual preferences was reduced, and multiple trials of one experimental condition increased the signal-to-noise ratio. The tunes were copied from commercially available CDs (see S1 Table and for a sound sample see S1 Sound, all stimuli are provided on request by the corresponding author) and have been used in previous studies [68,69,70]. (b) *Isochronous tones* were sequences of Shepard tones [71] (see S2 Sound sample, all stimuli are provided on request by the corresponding author). Shepard tones are complex tones that elicit the perception of a clear pitch class but not a clear pitch height. Each tone had a duration of 100 ms (see Fig 1) and a pitch class that was one semitone higher than the pitch class of the previous tone. Thus, the series of Shepard tones evoked the illusion of tones ascending along the chromatic scale. We used tones (and not simply clicks, or percussive sounds) so that both the *isochronous tones* and the *pleasant music* stimuli would contain pitch information that changed from tone to tone. By using Shepard tones instead of tones with unambiguous clear pitch, we avoided large interval jumps that would have been necessary to return to a lower register once sequences of ascending tones with unambiguous pitch reach very high pitch registers. Thus, the use of ascending Shepard tones established a musical tactus while keeping the melodic information to a minimum. (c) The third stimulus type consisted of the isochronous Shepard tones stimuli described in (b) overlaid with unpleasant *music-like noise*. This *music-like noise* was produced by editing excerpts of slow instrumental tunes copied from commercially available CDs (see S1 Table for list of tunes). Each of these excerpts was layered with a copy one semitone higher and a copy one tritone lower. All three versions of one excerpt were rendered as a single wav file (as in [69,72,73], pitch-shift and rendering was performed using *Cubase*, Steinberg, Hamburg, Germany), which resulted in a highly dissonant sound file. Then, this file was re-recorded backwards (to further reduce the musical quality, as well as increase the noise quality), and overlaid over the recording of the Shepard tones (see Fig 1 and S3 Sound sample, all stimuli are provided on request by the corresponding author). This process created highly unpleasant dissonant *music-like noise* stimuli with an isochronous pulse. Importantly, for each *pleasant music* stimulus, a corresponding *isochronous tones* sequence with identical tempo (number of bpm) and *music-like noise* stimulus including the *isochronous tones* sequence was created. Therefore, tempo (average number of bpm was 119) did not differ between the three stimulus types (see also Fig 1). All of the stimuli were edited to be 90 seconds long to avoid an overly long experimental session (recall that a variety of stimuli was used to avoid that personal preferences bias the results). Intensity (RMS power) was adjusted to be equal for all stimuli.

#### Procedure

Participants listened to the stimuli through headphones at a comfortable loudness of around 60 dB in supine position with closed eyes. The task was to focus attention on the stimuli and to tap the beat of the stimuli with the index finger of the right hand. This tapping allowed us to control whether participants paid equal attention to the three stimulus types, similar to a previous study [72]. Twenty-five consecutive trials were presented (see Fig 1): six trials with *pleasant music*, six trials with *isochronous tones*, six trials with unpleasant *music-like noise* as well as seven trials without sound (henceforth referred to as *silence* trials). The ordering of trials was pseudo-randomized; however, the first and last trials were *silence* trials. The stimuli were presented using *Presentation* (Neurobehavioral Systems Inc., Albany, CA, USA). After each trial, the subjects rated how pleasant they felt during the trial (i.e., during listening of the stimulus, or during resting in silence) on a six point Likert scale from 1 (“very pleasant”) to 6 (“very unpleasant”). Importantly, felt emotion was rated, instead of emotion perceived as expressed by the stimuli (i.e., participants were asked to rate their own emotional state, and not asked to make an assessment of the emotions expressed by the music). The duration of Experiment 1 was approximately 44 min.

#### Data acquisition

From each participant, a standard 12-lead rest electrocardiogram (ECG) was recorded with disposable electrodes in high resolution (sampling rate of 1000 Hz, bit-rate of 22) and stored in digital format. EDA was obtained with two Ag-AgCl electrodes placed on the palm of the third and fourth finger of the left hand. The electrodes applied square wave currents of 200 mV at 70 Hz. Due to artifacts, EDA data of only 72 subjects (33 female) were included in the data analysis. ECGs and EDA were recorded using *MREFA* amplifiers (Twente Medical Systems, Enschede, Netherlands).

#### Data analysis

Raw data analysis was performed with *MATLAB* (The Mathworks Inc., Natick, MA, USA). ECGs were 30 Hz low-pass filtered; artifacts were identified by visual inspection and excluded from further data analysis. The EDA signal was low-pass filtered with 8.5 Hz and downsampled to 20 Hz. ANS parameters were obtained with *MATLAB*-based open-source software: From the ECG, interbeat intervals, HR and HRV parameters (see Table 1) were computed using our in-house software package *Kardionoon* [74]. From the EDA data, the sum of amplitudes greater than .05 μS over the entire time of each trial was computed using *Ledalab* (www.ledalab.de). The statistics were performed using *PASW 18* (SPSS Inc., Chicago, IL, USA). The alpha level was set to .05, and two-sided hypotheses were tested. Valence (pleasantness) ratings and EDA data were analyzed with Friedman′s ANOVA and post-hoc Wilcoxon signed-rank tests with Bonferroni correction of the *p*-values (*p*-values of non-significant effects are provided without Bonferroni-correction, to avoid misinterpretations regarding beta-error estimation). HR and HRV data distribution was assessed visually and by Shapiro-Wilk tests. To account for skewed data (all parameters except the normalized units of low frequency HRV power, LF n.u.), decadic logarithmic transformation was performed and transformed data were analyzed. Repeated measures ANOVAs were computed to analyze HR and HRV across the experimental conditions (i.e., *pleasant music*, *isochronous tones*, *music-like noise*, and *silence* trials). Sphericity was assessed by Mauchly′s test, and correction was applied as indicated. Planned contrasts were designed to examine the main hypothesis that HR and HRV were different during the three stimulus types (i.e., *pleasant music*, *isochronous tones*, and *music-like noise)* compared to *silence*. Post-hoc analyses with Bonferroni correction of the *p*-values (*p*-values of non-significant effects are provided without Bonferroni-correction) were performed to compare results of *pleasant music* with *isochronous tones* as well as *pleasant music* with *music-like noise* (see Introduction of Experiment 1).

### Results

#### Valence ratings

Ratings of felt pleasantness differed significantly between experimental conditions: *χ* ^*2*^(3) = 189.3, *p* < .001, Kendall′s *W* = .83. As expected, on the Likert scale from 1 (“very pleasant”) to 6 (“very unpleasant”), listening to *pleasant music* (*Mdn* = 1.8) was rated more pleasant than resting in *silence* (*Mdn* = 2.4, *z* = -4.9, *p* < .001, *r* = -.4), more pleasant than listening to *isochronous tones* (*Mdn* = 4.1, *z* = -7.58, *p* < .001, *r* = -.61), and more pleasant than listening to *music-like noise* (*Mdn* = 4.7, *z* = -7.58, *p* < .001, *r* = -.61). Resting in *silence* was rated more pleasant than listening to *isochronous tones* (*z* = -7.5, *p* < .001, *r* = -.61) and listening to *music-like noise* (*z* = -7.56, *p* < .001, *r* = -.61). Listening to *isochronous tones* was rated more pleasant than listening to *music-like noise* (*z* = 4.75, *p* < .001, *r* = -.38) but less pleasant than the neutral value of 3.5 (*z* = -5.78, *p* < .001, *r* = -.47) All *p*-values of these singular comparisons were Bonferroni-corrected.

#### Heart rate

**Fig 2:**
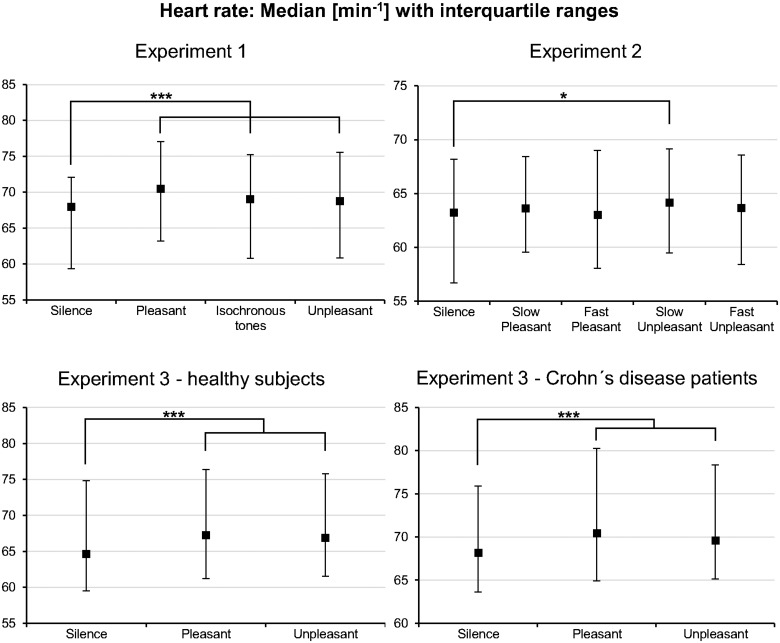
Heart rate results of all three Experiments. The acoustic stimuli generally increased the heart rate compared to silence. Although interquartile ranges were relatively wide, the effects were highly significant in Experiment 1 and 3. This indicates a larger sympathetic output during the acoustic stimuli. *Pleasant*: pleasant music; *Unpleasant*: music-like noise (Exp. 1 & 3) and unpleasant music (Exp. 2); *: *p* < .05; ***: *p* < .001.

Compared to *silence*, all stimulus types elicited an increase in HR: *F*(1.8, 135.29) = 63.64, *p* < .001, Greenhouse-Geisser *ε* = .6 (see Fig 2). The effect sizes were large (all *r*-values > .7, see S2 Table). Furthermore, HR was significantly higher during *pleasant music* than during *isochronous tones* and significantly higher during *pleasant music* than during *music-like noise* (see S2 Table).

#### Heart rate variability

**Fig 3:**
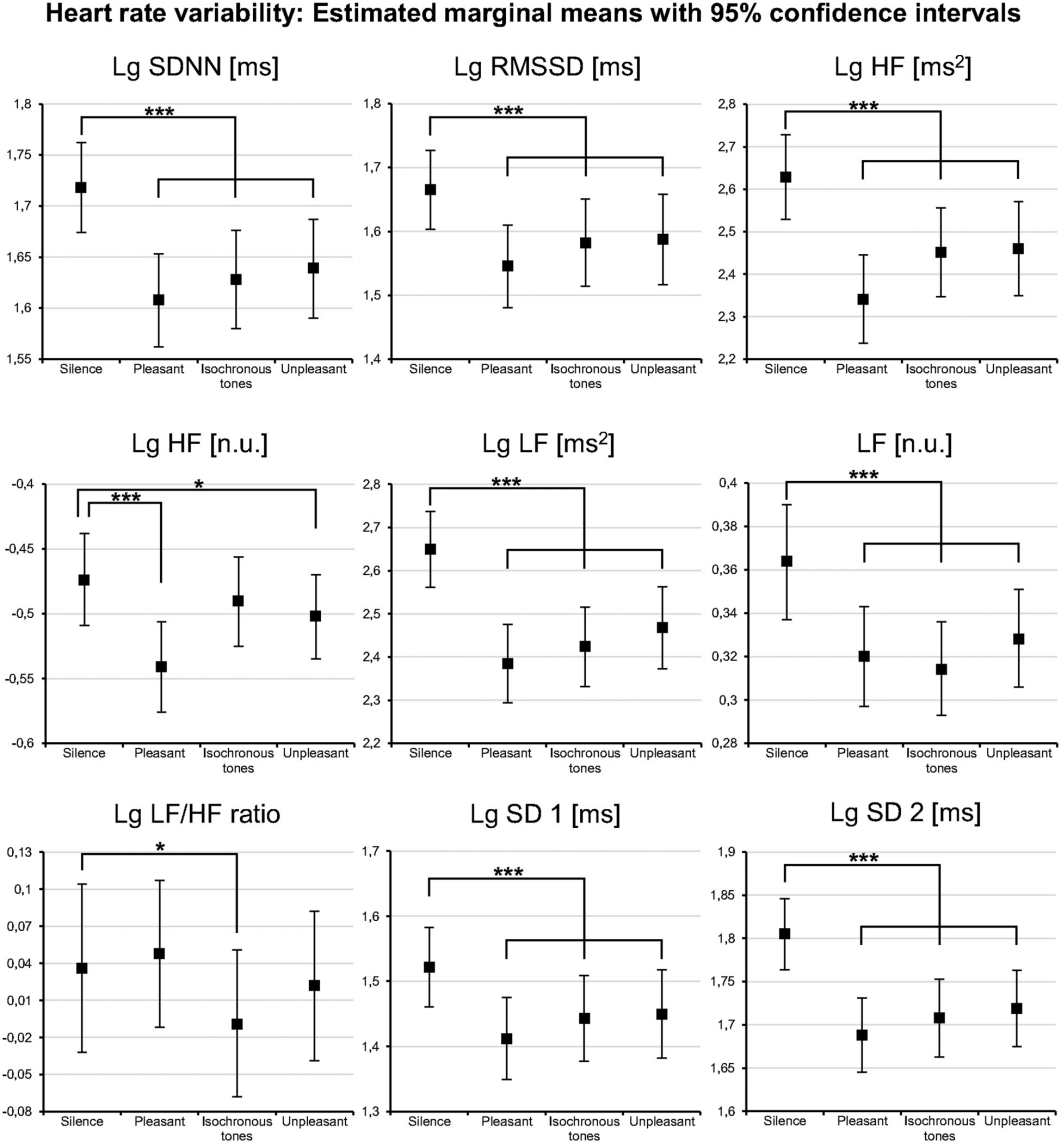
Heart rate variability results of Experiment 1. All acoustic stimuli decreased all heart rate variability parameters compared to silence except the LF/HF ratio. This is taken to indicate a reduced parasympathetic output during the acoustic stimuli. Notice that the effects with the isochronous tones, and music-like noise stimuli were qualitatively comparable to the pleasant music stimuli. *Lg*: indicates logarithmized data where transformation was applied; *Pleasant*: pleasant music; *Unpleasant*: music-like noise; *: *p* < .05; **: *p* < .01; ***: *p* < .001. For description of HRV parameters see Table 1.

Compared to *silence*, all stimulus types elicited a decrease in all HRV parameters (see Fig 3), except low frequency to high frequency power ratio (LF/HF). The effect sizes were generally large (see S3 Table). In addition, RMSSD, HF, HF n.u., and SD 1 (see Table 1 for description) were significantly lower during *pleasant music* than during *isochronous tones*, and lower during *pleasant music* than during *music-like noise* (see S4 Table).

#### Electrodermal activity

**Fig 4:**
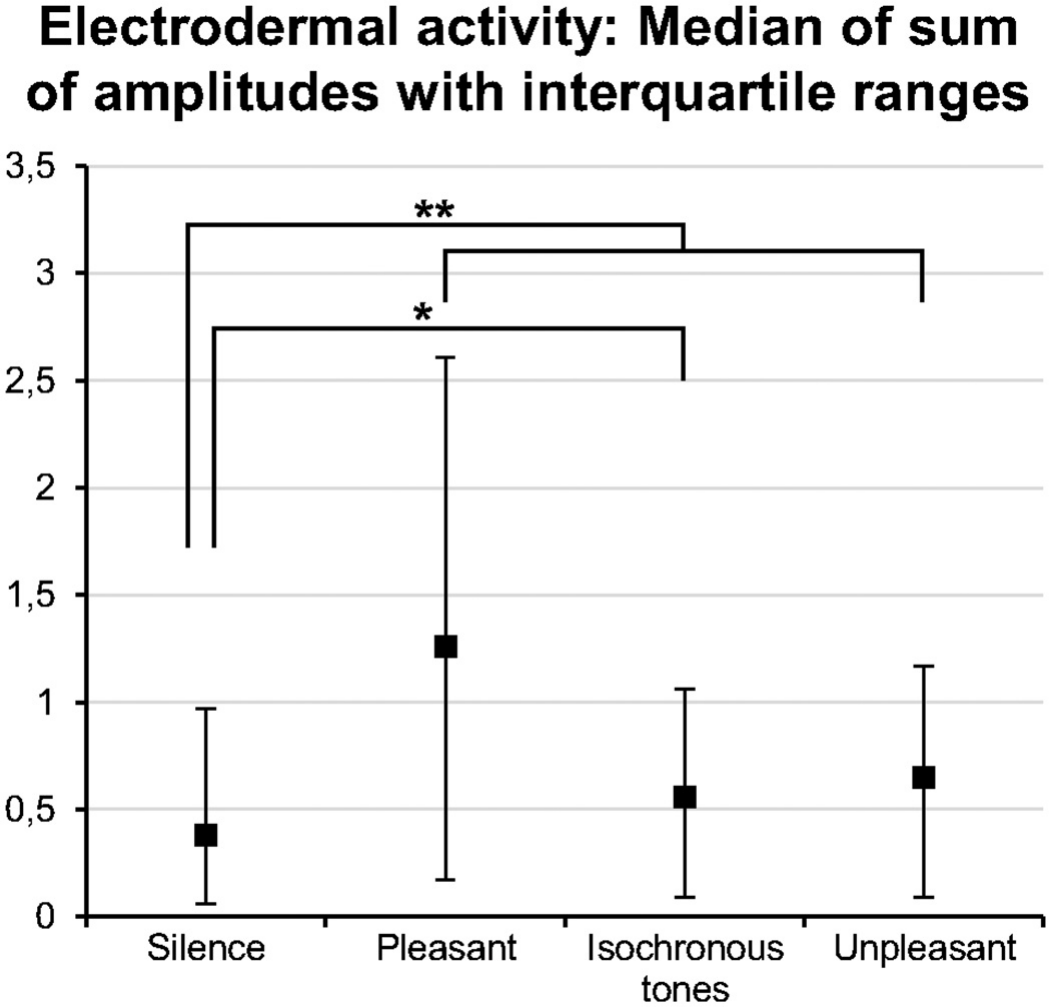
Electrodermal activity results of Experiment 1. All acoustic stimuli increased the sum of electrodermal activity amplitudes compared to silence. Although interquartile ranges were relatively wide, the effects were significant. This indicates a greater sympathetic output during the acoustical stimuli. *Pleasant*: pleasant music; *Unpleasant*: music-like noise; *: *p* < .05; **: *p* < .01.

Similarly to HR, all of the stimulus types elicited an increase in EDA compared to *silence*: *χ* ^*2*^(3) = 61.3, *p* < .001, Kendall′s *W* = .28 (see Fig 4). The effect sizes were small to moderate (see S5 Table). Moreover, *pleasant music* elicited significantly higher EDA than *music-like noise* (see S5 Table).

### Discussion

#### Effects of *pleasant music* and *isochronous tones* on HR, HRV, and EDA

Compared to *silence*, all stimulus types, i.e., *pleasant music*, *isochronous tones*, and *music-like noise*, elicited a similar pattern of autonomic effects: HR and EDA increased, whereas HRV decreased. Thus, both joyful music and even a simple tactus (i.e., simple isochronous sounds) elicited clear effects on peripheral indices of autonomic function. This outcome shows that even in the absence of known emotion-evoking mechanisms of music, such as emotional contagion, episodic memory, or musical expectancy violation [62], the presence of an isochronous auditory stimulus evokes clear effects on HR, HRV, and EDA. The results of the *isochronous tones* stimuli have to be interpreted with caution regarding music in general because they were artificially created as a tool to assess the isolated impact of a musical tactus on the ANS activity. However, these specific sounds allowed us to present isochronous stimuli with only minimal melodic information.

The increase in HR, and especially the increase in EDA (which is exclusively under sympathetic control), indicates that sympathetic activity increases during music listening (at least when the music has an average tempo of 119 bpm, as in this experiment; slower music will be addressed in Experiment 2). In addition, virtually all HRV parameters decreased; thus, parasympathetic activity is likely to decrease during music listening. Accordingly, the sympathovagal balance shifts towards sympathetic predominance. Experiments 2 and 3 will show that these effects are highly reliable.

#### Effects of emotional valence

The stimulus types evoked clear differences in felt pleasantness. *Pleasant music* evoked feelings of pleasantness, *isochronous tones* evoked feelings of minor unpleasantness, and *music-like noise* evoked feelings of distinct unpleasantness. Irrespective of these differences, all stimulus types elicited similar (not opposite) ANS effects when compared to silence (as described above). Nevertheless, *pleasant music* and unpleasant *music-like noise* also differed in their results. The increases in HR and EDA and the decreases in several HRV parameters (RMSSD, HF, HF n.u., and SD 1) were greater with *pleasant music* than with *music-like noise*. However, no significant differences in HR, HRV, or EDA were observed between *isochronous tones* and *music-like noise*, despite significantly different pleasantness ratings. Thus, the evoked valence differences did not systematically elicit significantly different ANS activity. Accordingly, the following experiments did not reproduce the differences observed between *pleasant music* and unpleasant *music-like noise* in this experiment.

## Experiment 2: The ANS response to different musical tempi

Experiment 2 was designed to replicate results of Experiment 1 and to assess the effects of different musical tempi (i.e., numbers of bpm) on ANS activity. Several previous studies investigated the effects of musical tempo on ANS activity; however, in all of these studies, stimuli were used that not only differed in tempo but also in emotional expression or musical genre. Thus, it was reported that higher musical tempo increases HR [25,75,76] and EDA [75,76] compared to lower tempo, but subjects listened to, e.g., slow melancholic classical music and fast exciting classical music [25,75], slow classical music and fast techno music [25], or slow tranquilizing pleasant classical music and fast heavy metal music perceived as arousing and unpleasant [76]. Consequently, the effects of tempo, valence, and arousal could not be disentangled in these studies, limiting the conclusions on unbiased effects of musical tempo on ANS activity. To clarify the specific effects of musical tempo, we used stimuli that differed only in tempo, i.e., the same musical pieces were presented in different tempi. Importantly, as results will show, subjective ratings of valence and arousal did not differ between slower and faster stimuli.

### Methods

#### Ethics statement

The study was approved by the ethics committee of the University of Leipzig. Written informed consent was obtained from all participants.

#### Subjects

Data were obtained from 30 healthy participants (17 female, aged 18 to 35 years) randomly selected from our institutional database of volunteers (see Experiment 1). As in Experiment 1, exclusion criteria comprised any history of somatic or psychiatric disease (including hearing disorders and diseases interfering with ANS activity such as diabetes mellitus or affective disorders).

#### Stimuli

Four types of acoustical stimuli were used: a slow version (90 bpm) of *pleasant music*, a fast version (120 bpm) of *pleasant music*, a slow version (90 bpm) of *unpleasant music*, and a fast version (120 bpm) of *unpleasant music*. To create the *pleasant music* stimuli of Experiment 2, the scores of 10 joyful instrumental tunes (see S6 Table) were translated into *Musical Instrument Digital Interface* (MIDI) format using *Cubase* (Steinberg Media Technologies, Hamburg, Germany). Then, each MIDI file was exported as a wav file with 90 bpm as well as 120 bpm using a digital piano synthesizer. Thus, pairs of slow and fast *pleasant music* stimuli (with the same musical content, i.e., the same notes played in different tempi) were created (for samples see S4 and S5 Sounds, all stimuli are provided on request by the corresponding author). However, the use of MIDI translation and digital instrument recording produced stimuli lacking the expressive timing and dynamics of the original musical pieces. *Unpleasant music* stimuli were electronically modified versions of these *pleasant music* files: Every *pleasant music* stimulus was layered with a copy pitch shifted one semitone higher and a copy pitch shifted one tritone lower (as in Experiment 1). All three versions of this *pleasant music* stimulus were rendered into a single wav file, creating highly dissonant sound files (*Cubase* was used for all steps of this process). This process created pairs of slow and fast *unpleasant music* stimuli from the slow and fast *pleasant music* stimuli (for samples see S6 and S7 Sounds, all stimuli are provided on request by the corresponding author). Other than in Experiment 1, the *unpleasant music* stimuli were presented without simultaneous Shepard tones because they already had exactly the same tempo as the pleasant stimuli. To avoid an overly long duration of the experimental session (the duration of Experiment 2 was already significantly longer than the duration of Experiment 1), no condition with *isochronous tones* was used in Experiment 2. All stimuli were edited to be 60 seconds long. Intensity (RMS power) was adjusted to be equal for all stimuli.

#### Procedure

As in Experiment 1, the participants listened to the stimuli through headphones at a comfortable loudness of around 60 dB in supine position with closed eyes. The task was to focus on the stimuli and to tap the beat with the right index finger. Forty-three consecutive trials were presented: 10 trials with slow *pleasant music*, 10 trials with fast *pleasant music*, 10 trials with slow *unpleasant music*, 10 trials with fast *unpleasant music*, and three *silence* trials without sound. To facilitate autonomic entrainment, frequent tempo changes were avoided and two blocks with all 20 stimuli of one tempo were presented. That is, one block consisted of 10 slow *pleasant music* stimuli and 10 slow *unpleasant music* stimuli, and the other block consisted of 10 fast *pleasant music* stimuli and 10 fast *unpleasant music* stimuli. Within these blocks, *pleasant music* and *unpleasant music* stimuli alternated. The ordering of the blocks was counterbalanced across participants. Before the first block, between the blocks, and after the second block, *silence* trials were presented. After each stimulus, subjects rated how pleasant they felt during listening on a nine point Likert scale from 1 (“very pleasant”) to 9 (“very unpleasant”) as well as how agitated they felt during listening on a nine point Likert scale from 1 (“very calm”) to 9 (“very agitated”). The duration of Experiment 2 was approximately 56 min.

#### Data acquisition

ECG recording was identical to Experiment 1. EDA was not measured in Experiment 2.

#### Data analysis

Analysis of felt pleasantness was identical to Experiment 1, and arousal data were analyzed likewise. ECG data analysis was identical to Experiment 1, however, due to artifacts, ECG data of only 29 participants were analyzed (17 female). To account for skewed data, all HRV parameters, except normalized units of low frequency power (LF n.u.), were transformed using the decadic logarithm prior to analysis. Planned contrasts of the repeated measures ANOVAs were designed to reproduce Experiment 1, i.e., whether HR and HRV changed as a function of the stimulus types (slow *pleasant music*, fast *pleasant music*, slow *unpleasant music*, fast *unpleasant music*) compared to *silence*. Post-hoc tests with Bonferroni correction of the *p*-values were used to analyze the effects of tempo (slow vs. fast) and valence (pleasant vs. unpleasant) on HR and HRV (*p*-values of non-significant effects are provided without Bonferroni-correction, to avoid misinterpretations regarding beta-error estimation).

### Results

#### Valence and arousal ratings

Ratings of felt pleasantness differed significantly between stimulus types: *χ* ^*2*^ (3) = 49.38, *p* < .001, Kendall′s *W* = .55. On the Likert scale from 1 (“very pleasant”) to 9 (“very unpleasant”), listening to *pleasant music* (*Mdn* = 2.4) was rated more pleasant than listening to *unpleasant music* (*Mdn* = 3.8). This outcome was the case for slow *pleasant music* (*Mdn* = 2.4) versus slow *unpleasant music* (*Mdn* = 3.7, *z* = -4.5, *p* < .001, *r* = -.58), as well as for fast *pleasant music* (*Mdn* = 2.3) versus fast *unpleasant music* (*Mdn* = 3.8, *z* = -4.47, *p* < .001, *r* = -.58). The *p*-values of these significant effects were Bonferroni-corrected. There were no differences in felt pleasantness between listening to the slow and fast versions of *pleasant music* (*z* = -1.3, *p* = .2), nor between listening to the slow and fast versions of *unpleasant music* (*z* = -.17, *p* = .88).

Ratings of felt arousal differed significantly between stimulus types: *χ* ^*2*^(3) = 23.41, *p* < .001, Kendall′s *W* = .26. On the Likert scale from 1 (“very calm”) to 9 (“very agitated”), listening to *unpleasant music* (*Mdn* = 3.0) was rated more arousing than listening to *pleasant music* (*Mdn* = 2.6). This outcome was the case for slow *unpleasant music* (*Mdn* = 3) versus slow *pleasant music* (*Mdn* = 2.6, *z* = -3.79, *p* = .002, *r* = -.49), as well as fast *unpleasant music* (*Mdn* = 3.1) versus fast *pleasant music* (*Mdn* = 2.7, *z* = -3.37, *p* = .002, *r* = -.43). The *p*-values of these significant effects were Bonferroni-corrected. However, there were no differences in felt arousal between listening to the slow and fast versions of *unpleasant music* (*z* = -1.36, *p* = .18), or between listening to the slow and fast versions of *pleasant music* (*z* = -1.27, *p* = .21).

#### Heart rate

Consistent with the results of Experiment 1, HR increased nominally with all stimulus types when compared to *silence* (see Fig 2). However, these changes in HR were very small, and in contrast to Experiment 1, they were not statistically significant: *F*(1.44, 40.2) = 1.29, *p* = .28, Greenhouse-Geisser *ε* = .36. Nevertheless, planned contrasts indicated that HR increased significantly during slow *unpleasant music* (see S7 Table). Importantly, the different tempi had no effect on HR (see S7 Table). Likewise, no differences in HR were observed between the *pleasant music* and *unpleasant music* stimuli (see S7 Table).

#### Heart rate variability

**Fig 5:**
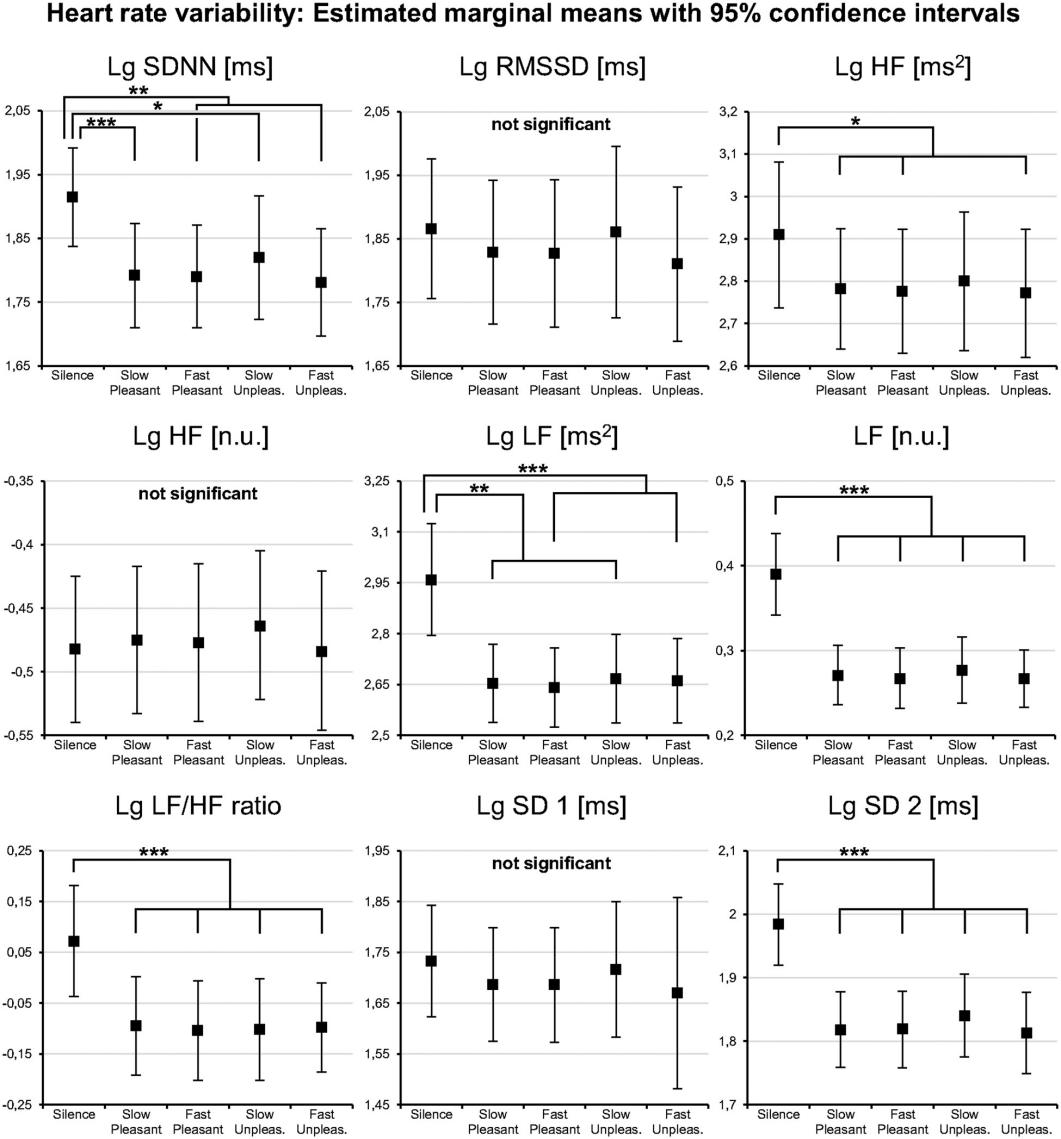
Heart rate variability results of Experiment 2. The acoustic stimuli decreased most of the heart rate variability parameters compared to silence, thus generally reproducing the results of Experiment 1. Note the similar results of the slow (90 bpm) and fast (120 bpm) versions of pleasant music and unpleasant music. This indicates that such a tempo difference does not entrain different heart rate variability. *Lg*: indicates logarithmized data where transformation was applied; *Pleasant*: pleasant music; *Unpleas*.: unpleasant music; *: *p* < .05; **: *p* < .01; ***: *p* < .001. For description of HRV parameters see Table 1.

Compared to silence, all stimulus types elicited a nominal decrease in all HRV parameters (see Fig 5), except normalized units of high frequency power (HF n.u.). Compared to Experiment 1, fewer HRV parameters decreased significantly (i.e., only SDNN, LF, LF n.u., LF/HF, and SD 2, see S8 Table and Table 1 for a description of HRV parameters). The effect sizes were moderate to high (see S8 Table). The different tempi had no effect on HRV (see S9 Table). Likewise, no differences were observed between the *pleasant music* stimuli and the *unpleasant music* stimuli (see S9 Table).

### Discussion

#### Effects of *pleasant music* and *unpleasant music* on HR and HRV

Consistent with Experiment 1, all of the stimulus types evoked—compared to *silence*—an increase in HR (albeit not statistically significant) and a decrease in HRV (although fewer HRV parameters showed significant changes than in Experiment 1). Concerning the sympathovagal balance, the results of Experiment 2 were less conclusive than Experiment 1, in which auditory stimuli evoked an increase of sympathetic activity (as indicated by an increase HR and EDA) and a decrease in parasympathetic activity (as indicated by a decrease in virtually all HRV parameters). The HRV parameters that decreased significantly in Experiment 2 (i.e., LF, LF n.u., LF/HF, and SD 2) are often interpreted as reflecting sympathetic contributions to HRV. Accordingly, this result would suggest a decrease in sympathetic activity instead of an increase. However, as specified in the General Discussion, the interpretation of HRV parameters is still a subject of debate and we refrain from a strict attribution of single HRV parameters to the sympathetic or parasympathetic system. Moreover, an actual decrease in sympathetic activity would have resulted in a decrease in HR, whereas an increasing trend was observed. Thus, we conclude that, consistent with Experiment 1, listening to music in Experiment 2 evoked a shift of the sympathovagal balance towards sympathetic activity.

The use of MIDI-derived stimuli without expressive timing and dynamics may be the reason for the less distinct results of Experiment 2 compared to Experiment 1. However, Experiment 1 showed that a tactus alone is sufficient to evoke highly significant ANS responses. The stimuli of Experiment 2 had a clear tactus and rhythm. Therefore, the smaller sample size, and perhaps the lower number of trials in Experiment 2 (1160 musical trials, compared to 1368 musical trials in Experiment 1) might have led to a lower statistical power, and thus to the different results of Experiments 1 and 2.

#### Effects of tempo and emotional valence

Felt pleasantness and arousal did not differ with tempo, i.e., between listening to slow and fast *pleasant music*, or between listening to slow and fast *unpleasant music*. Thus, we were able to study effects of tempo on the ANS activity without the results being biased by effects due to valence or arousal. Our data indicate that differences in tempo (90 vs. 120 bpm) between auditory stimuli—that are otherwise identical—do not result in different ANS activity. Specifically, neither HR nor HRV entrained with the slow and fast stimuli, although participants listened to one tempo for several minutes followed by another tempo for several minutes (recall that stimuli with the same tempo were presented in blocks). Therefore, our results challenge the notion that the tempo of the musical tactus (or “beat”) influences or entrains ANS activity [77,78], thus further challenging the notion that entrainment processes represent a basic mechanism of emotion induction by music [62,79]. This conclusion is somewhat limited by the fact that the difference in tempo was moderate (90 vs. 120 bpm). In fact, a previous study reported HR effects with a tempo difference of 55 vs. 136 bpm [25]. However, the musical stimuli used in that study [25] differed greatly in musical content (a tender tranquilizing versus an exciting arousing piece of classical music), and another study did not demonstrate HR effects with a tempo difference of 60 vs. 130 bpm [16], adding evidence to the conclusion that the role of musical tempo is somewhat marginal when arousal is controlled for.

Consistent with Experiment 1, *pleasant music* stimuli evoked greater feelings of pleasantness than *unpleasant music* stimuli. However, whereas in Experiment 1, HR and HRV differed significantly between *pleasant music* and unpleasant *music-like noise* (but not between *isochronous tones* and *music-like noise*), they did not differ between *pleasant music* and *unpleasant music* in Experiment 2. Thus, in our study pleasantness/unpleasantness evoked by musical stimuli did not exert a systematic effect on the ANS activity. Experiment 3 will further underline this notion.

## Experiment 3: The reliability of ANS responses to music and the effect of Crohn′s disease

Experiment 3 aimed to replicate the results of Experiments 1 and 2. That is, we expected significant ANS effects (as reflected in HR and HRV) in response to auditory stimuli compared to silence. Additionally, Experiment 3 aimed to clarify the mixed results of Experiments 1 and 2 concerning the impact of music-evoked pleasantness on the ANS activity. The same stimuli as in Experiment 1 were used (to guarantee that results can be optimally compared). Moreover, to examine the feasibility and efficacy of our stimuli for future clinical trials, Experiment 3 was performed with a group of healthy participants and a group of patients with Crohn′s disease (CD, a chronic inflammatory bowel disease). CD was chosen because the ANS is discussed as a factor in pathogenesis and a possible target for therapeutic intervention in chronic inflammation in general [80] and specifically in CD [81,82]. Furthermore, music could represent a therapeutic intervention targeting the ANS in CD, because previous studies demonstrated the successful clinical implementation of music in other clinical environments [52,83,84]. Another possible use of music in CD might be as a screening tool for autonomic dysfunction in CD. However, as reviewed below, studies of ANS activity in CD are sparse, the results are inconclusive, and additional research on the ANS in CD is needed [81]. Therefore, in Experiment 3, we (a) compared overall HR and HRV of CD patients with matched healthy participants, thus examining general or tonic ANS activity in CD; and (b) tested for differences in the ANS responses to the acoustical stimuli, thus examining ANS reactivity in CD. To describe why it is reasonable and imperative to investigate the ANS in CD, the next two sections will provide a brief overview over the available data of ANS activity in intestinal inflammation (the characteristic feature of CD) and CD.

### Intestinal inflammation and the ANS

In addition to the well-described clinical significance of the ANS in cardiovascular disease [85], affective disorders [8], and chronic pain [86], the ANS also regulates the immune system. For example, a cholinergic anti-inflammatory pathway has been described, which is a reflex arc that diminishes inflammation via the parasympathetic vagus nerve [87]. In intestinal inflammation, animal studies demonstrated a pivotal role of this pathway and therefore of the ANS. Specifically, parasympathetic activity is taken to protect against intestinal inflammation [88,89], whereas sympathetic activity appears to worsen intestinal inflammation [90]. Moreover, the affective system (and thus emotion) modulates these effects [91]. In humans, the significance of the ANS in intestinal inflammation was demonstrated in ulcerative colitis, an inflammatory bowel disease of the colon [92]. Multiple studies reported evidence of a shift of the autonomic balance towards sympathetic activity in ulcerative colitis [93,94,95,96,97,98]. Furthermore, pharmacological dampening of sympathetic activity decreased disease activity in ulcerative colitis [93,99].

### Crohn´s disease and the ANS

CD is a chronic inflammatory bowel disease characterized by relapsing mucosal inflammation of the digestive system, especially the small intestine and the colon [100]. However, additional extraintestinal inflammatory phenomena occur frequently [101], demonstrating the underlying systemic character of CD. Several studies have investigated ANS activity in CD patients, but the results are inconclusive. Studies reporting a high incidence of autonomic neuropathy in CD [102,103] are frequently cited as evidence of autonomic dysfunction in CD. However, the methods of these studies have been criticized [104]. Moreover, regarding HRV in CD, one study reported lower rest values of SDNN, RMSSD, LF, HF, and SD 1 (see Table 1 for description) in CD compared to healthy subjects [105]. However, other studies did not demonstrate differences in HRV at rest between CD patients and healthy subjects [96,97]. To our knowledge, there is lack of therapeutic trials targeting the ANS in CD (whereas several trials were conducted in ulcerative colitis, see section above), and neither music nor other auditory stimuli have been used so far to study the ANS activity in CD. However, as Experiments 1 and 2 have demonstrated, music and music-like stimuli are capable of inducing medium to large effects in ANS parameters like HR and HRV. Therefore, music might represent a potential diagnostic tool to screen for ANS dysfunction [50] and, moreover, might also have therapeutic potential.

### Methods

#### Ethics statement

The study was approved by the ethics committee of the University of Leipzig. Written informed consent was obtained from all participants.

#### Subjects

Data were obtained from 19 CD patients and 32 healthy participants (for comparative epidemiologic data of both groups of subjects, see Table 2). CD patients were treated as outpatients at the time of the study. Diagnosis of CD had been established according to endoscopic and microscopic criteria [106]. Clinical data of CD patients are provided in Table 3. Exclusion criteria for CD patients were additional conditions (e.g., diabetes mellitus) or medication (e.g., beta blockers) known to affect the ANS or the HR and HRV measurements.

**Table 2:** Characteristics of participants in Experiment 3.

	CD patients	Healthy participants	Statistics
Gender	13 female (68%)	20 female (63%)	*χ* ^*2*^(1) = .18, *p* = .77
Age	*Mdn* = 39 years, *IQR* = 25	*Mdn* = 38.5 years, *IQR* = 25	*Z* = .27, *p* = .998
Body mass index**	*M* = 21.7 kg/m^2^, *SEM* = .76	*M* = 25.4 kg/m^2^, *SEM* = .77	*t*(49) = -3.22, *p* = .002
Weekly physical activity	*Mdn* = .5 hours, *IQR* = 6	*Mdn* = 3.3 hours, *IQR* = 5.5	*Z* = .95, *p* = .19
Nicotine abuse	3 smokers (16%)	11 smokers (34%)	*Χ* ^*2*^(1) = 2.07, *p* = .2
Time of measurement	*Mdn* = 2 pm, *IQR* = 6 hours	*Mdn* = 5 pm *IQR* = 7 hours	*Z* = 1.13, *p* = .07

**: *p* < .01;

*CD*: Crohn′s disease; *Mdn*: median; *IQR*: interquartile range; *M*: mean; *SEM*: standard error of mean; *χ* ^*2*^: Pearson′s Chi-Square test; *Z*: Kolmogorov-Smirnov-Z test.

**Table 3:** Clinical characteristics of Crohn′s disease patients of Experiment 3.

	Descriptive statistics
Duration of disease	*Mdn* = 8 years, *IQR* = 10
Crohn′s disease activity index CDAI^1^	*M* = 128, *SD* = 28 (n = 14)
Prior surgical therapy of Crohn′s disease	*n* = 10 (53%)
Current use of glucocorticoids or immunosuppressives	*n* = 14 (74%)
Current use of complementary medicine	*n* = 3 (16%)
Current extraintestinal manifestations	*n* = 3 (16%)

*Mdn*: median; *IQR*: interquartile range; *M*: mean; *SD*: standard deviation;

^1^ CDAI above 150 indicates active disease; CDAI below 150 indicates disease in remission.

Healthy participants were recruited to match CD patients from our institutional database of volunteers (see Experiment 1). As in Experiment 1 and 2, exclusion criteria comprised any history of somatic or psychiatric disease (including hearing disorders and diseases interfering with ANS activity such as diabetes mellitus or affective disorders). Healthy participants had a significantly higher body mass index than the CD patients but were otherwise comparable in other factors known to modulate ANS activity (see Table 2).

#### Stimuli

*Pleasant music* stimuli (i.e., excerpts of joyful music from different genres) and *music-like noise* stimuli (i.e., highly dissonant sounds with additional scales of isochronous tones) of Experiment 1 were used. To focus in Experiment 3 on the ANS effects of music and music-evoked pleasantness, and to keep the experimental duration short for the patients, *isochronous tones* stimuli were not used.

#### Procedure

The experimental procedure and tasks were similar to Experiment 1 (see Fig 1). Seventeen trials were presented, encompassing six *pleasant music* stimuli, six *music-like noise* stimuli, and five *silence* trials without sound. As in Experiment 1, the ordering of trials was pseudo-randomized, with the first and last trials being *silence* trials. The subjects rated how pleasant they felt during the trial on a six point Likert scale from 1 (“very pleasant”) to 6 (“very unpleasant”). The duration of Experiment 3 was approximately 32 min.

#### Data acquisition

ECG recording was identical to Experiments 1 and 2. EDA was not measured in Experiment 3.

#### Data analysis

Analysis of pleasantness ratings was identical to Experiments 1 and 2. To compare ratings of both groups of participants, Kolmogorov-Smirnov-Z tests were computed instead of Wilcoxon signed-rank tests due to the smaller sample sizes. Raw ECG data processing was identical to Experiments 1 and 2. To account for skewed data, HR and all HRV parameters, except normalized units of low frequency power (LF n.u.), were transformed using the decadic logarithm prior to analysis. Mixed-design ANOVAs were computed for the comparison of CD patients with healthy participants. Planned contrasts were designed to test whether HR and HRV changed as a function of the stimulus types (*pleasant music* and *music-like noise*) compared to *silence*. Post-hoc analyses with Bonferroni correction of the *p*-values were performed to compare HR and HRV between *pleasant music* and *music-like noise* (*p*-values of non-significant effects are provided without Bonferroni-correction, to avoid misinterpretations regarding beta-error estimation).

### Results

#### Valence ratings

Consistent with the results of Experiment 1 and 2, the ratings of felt pleasantness of both groups of participants differed significantly between experimental conditions: *χ* ^2^(2) = 64.23, *p* < .001, Kendall′s *W* = .63. On the Likert scale from 1 (“very pleasant”) to 6 (“very unpleasant”), listening to *pleasant music* (*Mdn* = 2) was rated as pleasant as resting in *silence* (*Mdn* = 2, *z* = -.12, *p* = .91) and both were rated more pleasant than listening to *music-like noise* (*Mdn* = 4.5, for *pleasant music z* = -6.11, *p* < .001, *r* = -.6, and for *silence z* = -6.13, *p* < .001, *r* = -.61). CD patients and healthy participants did not differ in their felt pleasantness ratings. This was the case for listening to *pleasant music* (CD patients *Mdn* = 2.2, healthy participants *Mdn* = 1.9, *Z* = 1.18, *p* = .03, Bonferroni corrected alpha level was .017), listening to *music-like noise* (CD patients *Mdn* = 4.8, healthy participants *Mdn* = 4.3, *Z* = 1.07, *p* = .12), and resting in *silence* (CD patients *Mdn* = 2, healthy participants *Mdn* = 1.9, *Z* = .64, *p* = .49).

#### Heart rate

Replicating the results of Experiment 1, an increase in HR was elicited by both stimulus types: *F*(1.79, 87.93) = 28.89, *p* < .001, Huynh-Feldt *ε* = .9 (see Fig 2). That is, compared to *silence* (*Mdn* = 66.9 min^-1^), HR was higher with *pleasant music* (*Mdn* = 69 min^-1^, *F*(1, 49) = 42.07, *p* < .001, *r* = .68) as well as *music-like noise* (*Mdn* = 68.6 min^-1^, *F*(1, 49) = 43.7, *p* < .001, *r* = .68). No differences in HR were observed between *pleasant music* and *music-like noise* (*p* = .03, Bonferroni-corrected alpha level was .017). Furthermore, HR was not systematically different in CD patients compared to healthy participants (group main effect: *F*(1, 49) = 1.03, *p* = .32), and the groups did react equally to the three experimental conditions (interaction effect: *F*(1.79, 87.93) = .87, *p* = 41, see HR distribution patterns in Fig 2).

#### Heart rate variability

**Fig 6:**
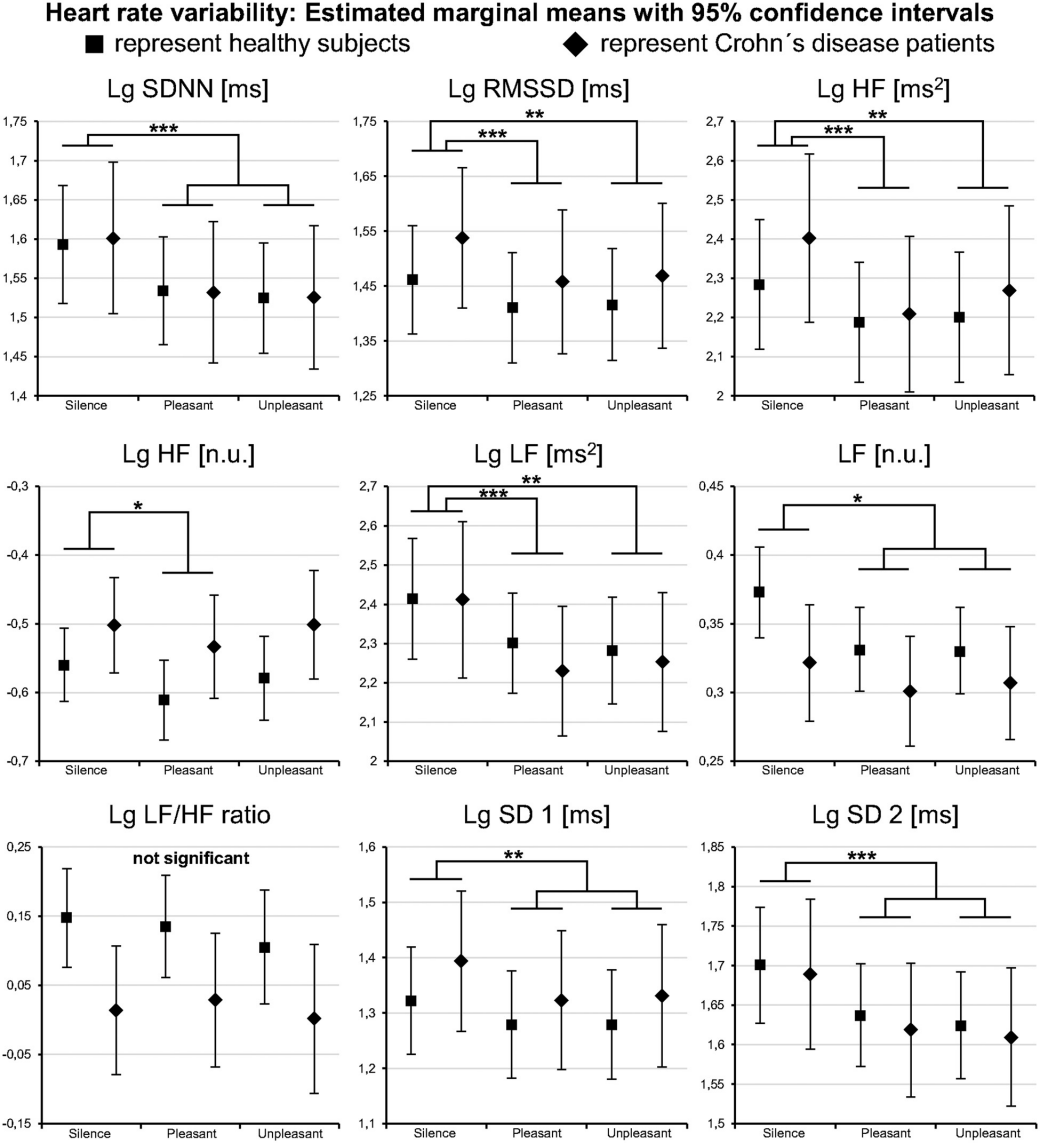
Heart rate variability results of Experiment 3. The acoustic stimuli decreased all heart rate variability parameters compared to silence except the LF/HF ratio, thus reproducing the results of Experiment 1 and 2. Note the generally similar results of healthy subjects and Crohn′s disease patients. This demonstrates that Crohn′s disease is not per se characterized by a different autonomic tone or autonomic reactivity. *Lg*: indicates logarithmized data where transformation was applied; *Pleasant*: pleasant music; *Unpleasant*: music-like noise; *: *p* < .05; **: *p* < .01; ***: *p* < .001. For description of HRV parameters see Table 1.

Replicating the results of Experiments 1 and 2, both stimulus types—compared to *silence*—elicited a significant decrease in all HRV parameters (see Fig 6), except normalized units of high frequency power (HF n.u.) and low frequency to high frequency power ratio (LF/HF). The effect sizes were moderate to large (see S10 Table). Comparing the results of *pleasant music* with *music-like noise*, HRV differed only in HF n.u. (see S11 Table). Between CD patients and healthy participants, no systematic HRV differences were observed (see S10 Table). Moreover, both groups demonstrated equal HRV reactions across the three experimental conditions (see HRV distribution patterns in Fig 6 and S10 Table).

### Discussion

#### Effects of joyful music on HR and HRV

Experiment 3 replicated the ANS responses elicited by the *pleasant music* stimuli in Experiments 1 and 2; specifically, HR increased and HRV decreased compared to resting in silence. Thus, listening to joyful music (as is listening to *music-like noise*, see next section) is characterized by a shift of the autonomic balance towards sympathetic activity, and the similarity of the results of Experiments 1 and 3 demonstrates the reliability of these effects. This outcome is not surprising because, as the *isochronous tones* stimuli of Experiment 1 demonstrated, a tactus (that was present in all our *pleasant music* stimuli) is responsible for a large share of the ANS effects of music.

#### Effects of emotional valence

Experiment 3 again produced clear differences in felt pleasantness: *Pleasant music* stimuli evoked feelings of pleasantness and *music-like noise* stimuli evoked feelings of unpleasantness. Despite this unambiguous effect on emotional valence, HR and HRV did not differ between the stimulus types except in a single HRV parameter (HF n.u.). This outcome substantiates the notion that un/pleasantness evoked by a musical stimulus does not systematically contribute to the ANS effects evoked by this musical stimulus, at least not with regard to the measures used in the present study. Another result of Experiment 3 supports this notion: *silence* trials evoked feelings of pleasantness equal to the *pleasant music* stimuli, nevertheless, clear differences in HR and all HRV parameters (except LF/HF) were demonstrated between both conditions (see paragraph above and S10 Table). Therefore, the presence of a musical stimulus with rhythmic features per se (e.g., a tactus, see discussion of isochronicity in Experiment 1 and paragraph above) appears to be a major factor responsible for ANS effects of music. Thus, our results indicate that the evocation of emotion is not a necessary condition to elicit ANS responses with music.

#### ANS activity in CD

All CD patients tolerated the experimental procedure well. Despite the elicitation of significant emotional and autonomic effects, no onset or worsening of symptoms was reported. Therefore, music is safe and—given the significant ANS reactions—also effective to assess ANS activity in CD. However, the results of Experiment 3 do not reflect autonomic dysfunction in CD. HR and HRV were not systematically different in CD patients, and the experimental conditions did not evoke different autonomic reactions in CD patients compared to healthy participants. Thus, physiological tonic sympathovagal balance and ANS reactivity to musical stimuli were preserved. Limiting this conclusion is the small number of CD patients studied. Thus, the statistical power of Experiment 3 might have been too small to detect minor effects (e.g., the group effect for LF/HF ratio was *p* = .06, see S10 Table). Furthermore, autonomic dysfunction, as reported for CD in some studies [102,103,105], may be prevalent only in subgroups of CD patients (e.g., patients with CD in remission that are treated with immunosuppressive drugs [105]). Therefore, additional research on ANS activity in CD is needed and should include predefined subgroups of CD patients, e.g., different levels of disease activity.

## General Discussion

According to our experimental rationale, this study tested (a) whether joyful music, compared to resting in silence, has any reliable effects on ANS activity, (b) whether a musical tactus (i.e., isochronous sound pulses) is sufficient to elicit similar effects, (c) whether emotional valence and musical tempo are factors that modulate these effects, and (d) whether ANS responses to music differ between patients with CD and healthy subjects. We were able to address all of these aspects, and the following sections will, in summary, discuss the results of all three experiments.

### The effects of joyful music on ANS activity

Table 4 shows the HR and HRV results of the *pleasant music* stimuli of all three experiments (compared to silence). Our data demonstrate clear effects of listening to joyful music on the ANS activity. First, HR consistently increased during music listening (although the increase was statistically not significant in Experiment 2). Second, the majority of analyzed HRV parameters consistently decreased during music listening (see Table 4). The results were inconsistent across the three experiments only for normalized units of high frequency power (HF n.u.) and low frequency to high frequency power ratio (LF/HF). In addition, the similarity of the results of Experiments 1 and 3 indicates that these effects in HR and HRV are highly reliable across a variety of music from different genres (see S1 Table). The predominantly moderate to large effect sizes (see Table 4) demonstrate the efficacy of music to elicit ANS effects, and indicate the large share in variability of ANS activity explained by the music stimuli. Several previous studies did not demonstrate effects of music on HR [39,40,41,42,43,44,45] and HRV [41,58,59]. However, our results indicate that music does evoke an increase in HR and a decrease in HRV. Moreover, the less pronounced results of Experiment 2 (with *n* = 30 participants) compared to Experiments 1 and 3 (with *n* = 76 and *n* = 51 participants, respectively) indicate that insufficient statistical power may explain some of the missing results in previous studies comparing effects of music to baseline values (none of which had more than 50 participants). We also presented a variety of experimenter-selected music pieces (in contrast to studies that used participant-selected music [28,39], or that presented only one piece of music per condition [30,32,41]). This strengthens the generalizability of our conclusions concerning joyful music, because the impact of individual taste in music can be considered small in our study.

**Table 4:** Summary of heart rate and heart rate variability effects of pleasant music compared to silence.

Parameter	Consistent result across all Experiments	Experiment 1	Experiment 2: slow	Experiment 2: fast	Experiment 3
HR	↑	↑ *** ‡‡‡	↑ *n.s.*	↑ *n.s.*	↑ *** ‡‡‡
SDNN	↓	↓ *** ‡‡‡	↓ *** ‡‡‡	↓ ** ‡‡‡	↓ *** ‡‡‡
RMSSD	↓	↓ *** ‡‡‡	↓ *n.s.*	↓ *n.s.*	↓ *** ‡‡
HF	↓	↓ *** ‡‡‡	↓ * ‡‡	↓ * ‡‡	↓ *** ‡‡‡
HF n.u.	inconsistent	↓ *** ‡‡	↑ *n.s.*	↑ *n.s.*	↓ * ‡‡
LF	↓	↓ *** ‡‡‡	↓ ** ‡‡‡	↓ *** ‡‡‡	↓ *** ‡‡
LF n.u.	↓	↓ *** ‡‡	↓ *** ‡‡‡	↓ *** ‡‡‡	↓ * ‡‡
LF/HF	inconsistent	↑ *n.s.*	↓ *** ‡‡‡	↓ *** ‡‡‡	↓ *n.s.*
SD°1	↓	↓ *** ‡‡‡	↓ *n.s.*	↓ *n.s.*	↓ ** ‡‡
SD°2	↓	↓ *** ‡‡‡	↓ *** ‡‡‡	↓ *** ‡‡‡	↓ *** ‡‡

^↑^: increase compared to silence values;

^↓^: decrease compared to silence values.

Significance level:

*: *p <* .05;

**: *p* < .01;

***: *p* < .001;

^*n*.*s*.^: not significant.

Effect size:

^‡‡^: *r* > .3 (indicates medium effect);

^‡‡‡^: *r* > .5 (indicates large effect).

Merging the results of the ANS parameters allows one to draw conclusions on the sympathovagal balance. The increase in HR, the increase in EDA (that was measured only in Experiment 1), and the decrease in HRV demonstrate that listening to joyful music evokes a shift towards sympathetic activity compared to resting in silence. This outcome represents a more conclusive body of evidence concerning the sympathovagal balance than the interpretation of single HRV parameters as representing sympathetic or parasympathetic activity (as in previous studies [25,34,54]). These HRV parameter labels are based upon initial physiological studies on HRV and were included in guidelines on HRV measurement [46]. However, due to a large body of new evidence, criticism of these interpretations has arisen, and the accuracy of autonomic interpretation of HRV parameters has been questioned [48]. For example, simply equating low frequency power (LF) with sympathetic activity does not seem to be valid. Consequently, the low frequency to high frequency power ratio (LH/HF), often claimed as representative of sympathovagal balance, is compromised. Considering these and other issues [48], we refrain from further conclusions on the basis of single HRV parameters. One might argue that the reason for the shift towards sympathetic activity that is elicited by joyful music compared to resting in silence represents an arousal reaction evoked by the beats of the acoustical stimulation [13,107]. This adds evidence to the not yet conclusively answered question as to whether listening to music generally increases or decreases HR and HRV compared to resting in silence (see Introduction).

### The effects of isochronicity on ANS activity

Beyond a simple description of music-evoked ANS effects, as elaborated on above, the scope of this study was to examine the factors responsible for these effects. Therefore, *isochronous tones* stimuli were presented in Experiment 1, representing a simple musical tactus. Table 5 summarizes the HR and HRV results of these stimuli compared to resting in silence together with the results of the *music-like noise* and *unpleasant music* stimuli. *Isochronous tones* elicited ANS effects that were comparable to the effects evoked by *pleasant music* (see Table 4): HR and EDA increased and all HRV parameters decreased. For the first time, this demonstrates that even a simple tactus is sufficient to evoke considerable responses in ANS activity (note the large effect sizes in Table 5). Thus, a large share of the ANS effects of music can be explained by the music′s isochronicity, i.e., the presence of a tactus or “beat”. However, significant differences in the strength of the HR and HRV reactions were observed between *isochronous tones* and *pleasant music*, i.e., HR increased less and HRV decreased less with *isochronous tones* compared to *pleasant music* (see S4 Table). Therefore, additional factors of joyful music that contribute to the ANS response have to be taken into consideration, such as emotional effects due to emotional contagion, or due to any other mechanism through which music can evoke emotions [62]. The chromatic scale of the Shepard tones and the artificial sound of the isochronous tones stimuli used in this study may have been a reason for the elicitation of moderate unpleasantness during listening to these stimuli (see valence ratings of Experiment 1). Therefore, we propose for future studies to consider the use of isochronous stimuli that elicit as little emotion as possible, in order to further investigate the role of a musical tactus for music-evoked ANS effects.

**Table 5:** Summary of heart rate and heart rate variability effects of isochronous tones, music-like noise, and unpleasant music compared to silence.

Parameter	Consistent result across all Experiments	Experiment 1 Isochronous tones	Experiment 1 Music-like noise	Experiment 2 Slow unpleasant music	Experiment 2 Fast unpleasant music	Experiment 3 Music-like noise
HR	↑	↑ *** ‡‡‡	↑ *** ‡‡‡	↑ * ‡‡	↑ n.s.	↑ *** ‡‡‡
SDNN	↓	↓ *** ‡‡‡	↓ *** ‡‡‡	↓ ** ‡‡	↓ ** ‡‡‡	↓ *** ‡‡‡
RMSSD	↓	↓ *** ‡‡‡	↓ *** ‡‡‡	↓ n.s.	↓ n.s.	↓ ** ‡‡
HF	↓	↓ *** ‡‡‡	↓ *** ‡‡‡	↓ n.s.	↓ * ‡‡	↓ ** ‡‡
HF n.u.	Inconsistent	↓ n.s.	↓ * ‡	↑ n.s.	↓ n.s.	↓ n.s.
LF	↓	↓ *** ‡‡‡	↓ *** ‡‡‡	↓ ** ‡‡‡	↓ *** ‡‡‡	↓ ** ‡‡
LF n.u.	↓	↓ *** ‡‡	↓ *** ‡‡	↓ *** ‡‡‡	↓ *** ‡‡‡	↓ * ‡‡
LF/HF	↓	↓ * ‡	↓ n.s.	↓ *** ‡‡‡	↓ *** ‡‡‡	↓ n.s.
SD°1	↓	↓ *** ‡‡‡	↓ *** ‡‡‡	↓ n.s.	↓ n.s.	↓ ** ‡‡
SD°2	↓	↓ *** ‡‡‡	↓ *** ‡‡‡	↓ *** ‡‡‡	↓ *** ‡‡‡	↓ *** ‡‡

^↑^: increase compared to silence values;

^↓^: decrease compared to silence values.

Significance level:

*: *p <* .05;

**: *p* < .01;

***: *p* < .001;

^*n*.*s*.^: not significant.

Effect size:

^‡^: *r* > .1 (indicates small effect);

^‡‡^: *r* > .3 (indicates medium effect);

^‡‡‡^: *r* > .5 (indicates large effect.

### The effects of music-evoked emotional valence and musical tempo on ANS activity

One factor commonly reported to contribute to music-evoked ANS effects is music-evoked emotion [62]. To study this factor, we presented joyful *pleasant music* stimuli (reliably eliciting pleasant feelings) as well as deliberately unpleasant *music-like noise* stimuli (reliably eliciting less pleasant feelings than *pleasant music*). The differences between the *pleasant music* stimuli and *music-like noise* stimuli in Experiment 1 at first implied that emotional valence may indeed play a role in music-evoked ANS effects. However, both Experiments 2 and 3 failed to demonstrate effects of pleasantness (whereas the ANS effects compared to *silence* were clearly replicated, see Tables 4 and 5). Thus, musical stimuli are sufficient to evoke a broad spectrum of emotional valence; however, these feelings of un/pleasantness do not contribute systematically to the ANS activity changes elicited by these stimuli. Nevertheless, music-evoked emotion may influence the ANS response in other settings, such as listening to music that has a biographic or aesthetic importance to the individual listener (as it is commonly used in studies examining “chills” evoked by music [28,33,108,109]) or during the combination of auditory stimulation with visual stimulation [110] (e.g., in operas [33] or film with music). Therefore, future research has to determine the factors and the necessary thresholds to surpass for emotional involvement to significantly play a role in music-evoked ANS responses.

Another factor commonly reported to determine music-evoked ANS effects is the musical tactus (or “beat”). According to theories of entrainment [62,63,65], internal autonomic processes synchronize to the tactus and thus tempo of the music. The remarkable effects of simply perceiving isochronous sounds (as described above) demonstrate the pivotal role of the tactus of music in ANS responses to music. In Experiment 2, we tested whether different musical tempi of a tactus actually entrain HR and HRV. The stimuli used to examine this effect in Experiment 2 presented—for the first time—different tempi to the listener without inducing concurrent, and thus confounding, emotional effects. However, no differences were observed between the slow (90 bpm) and the fast (120 bpm) stimuli. Therefore, changes in the musical tempo per se (without differences in arousal) do not evoke changes in HR or HRV. Thus, the abovementioned entrainment processes remain speculative. To test the validity of these conclusions, future studies are necessary to expand the body of data on musical tempo and ANS responses. For example, larger or smaller tempo differences may exert a greater modulation of internal autonomic processes.

### The impact of CD on music-evoked ANS effects

Music and music-like sounds are effective and safe to study the ANS in CD. According to the results of Experiment 3, the ANS activity in CD patients does not differ from healthy subjects. Thus, the ANS may be less involved in processes of disease in CD as suspected [81]. However, future research is necessary to broaden the evidence on the ANS activity in CD (e.g., investigating different clinical subgroups of CD patients).

## Conclusions

Listening to joyful pleasant music (compared to resting in silence) reliably evoked an increase in HR and a decrease in HRV, taken to indicate a shift of the sympathovagal balance towards sympathetic activity. However, simply a tactus (i.e., isochronous sound pulses) was sufficient to elicit similar effects. Thus, isochronicity of music was a major factor of the music-evoked ANS effects. However, the tempo of the tactus did not modulate the music-evoked ANS effects (given comparable emotional arousal across tempi). Emotional valence evoked by the music did not systematically influence ANS activity in this study. Moreover, music-evoked ANS activity of CD patients did not differ from that of healthy subjects.

## Supporting Information

S1 Dataset Experimental data of Experiment 1. (XLSX)

S2 Dataset Experimental data of Experiment 2. (XLSX)

S3 Dataset Experimental data of Experiment 3. (XLSX)

S1 Sound Excerpt of Suite No. 2 in B minor, BWV 1067, 7. Badinerie by Johann Sebastian Bach (pleasant music).
(MP3)

S2 Sound Shepard tones with 124 beats per minute (isochronous tones). (MP3)

S3 Sound Processed excerpt of Symphony No. 5, 4. Adagietto by Gustav Mahler layered with Shepard tones with 124 beats per minute (music-like noise).
(MP3)

S4 Sound Excerpt of The Easy Winners by Scott Joplin (slow pleasant music, 90 beats per minute). (MP3)

S5 Sound Excerpt of The Easy Winners by Scott Joplin (fast pleasant music, 120 beats per minute). (MP3)

S6 Sound Processed excerpt of The Easy Winners by Scott Joplin (slow unpleasant music, 90 beats per minute). (MP3)

S7 Sound Processed excerpt of The Easy Winners by Scott Joplin (fast unpleasant music, 120 beats per minute). (MP3)

S1 Table Pleasant music and music-like noise stimuli of Experiment 1 and 3. (DOCX)

S2 Table Heart rate results of Experiment 1. (DOCX)

S3 Table Heart rate variability results of Experiment 1. ANOVA main effects and planned comparisons.
(DOCX)

S4 Table Heart rate variability results of Experiment 1. ANOVA post-hoc analyses, mean differences of estimated marginal means [95% confidence intervals of mean difference].
(DOCX)

S5 Table Electrodermal activity results of Experiment 1. (DOCX)

S6 Table Pleasant music stimuli of Experiment 2. (DOCX)

S7 Table Heart rate results of Experiment 2. (DOCX)

S8 Table Heart rate variability results of Experiment 2. ANOVA main effects and planned comparisons.
(DOCX)

S9 Table Heart rate variability results of Experiment 2. ANOVA post-hoc analyses, mean differences of estimated marginal means [95% confidence intervals of mean difference], *p*-values Bonferroni-corrected.
(DOCX)

S10 Table Heart rate variability results of Experiment 3. ANOVA main effects, planned contrasts, group effects, and interaction effects.
(DOCX)

S11 Table Heart rate variability results of Experiment 3. ANOVA post-hoc analyses, mean differences of estimated marginal means [95% confidence intervals of mean difference].
(DOCX)
