# Supplementary material for: Autonomic Effects of Music in Health and Crohn's Disease: The Impact of Isochronicity, Emotional Valence, and Tempo
Source: PLoS One. 2015 May 8;10(5):e0126224. doi: 10.1371/journal.pone.0126224 (PMC4425535; doi:10.1371/journal.pone.0126224)
Supplement: S1 Table — (DOCX) [file pone.0126224.s011.docx]

**S1 Table. Pleasant music and music-like noise stimuli of Experiment 1 and 3.**

| Composer | Title | Style | Beats per minute |
| --- | --- | --- | --- |
| ***Pleasant music* stimuli** | | | |
| Antonio Vivaldi | Concerto grosso in G major, RV 310, 3. Allegro | Baroque | 73 |
| Jonathan Richman, Earl Zero | Egyptian Reggae | Reggae-Pop | 105 |
| W.C. Handy | St. Louis Blues | Blues | 117 |
| Johann Sebastian Bach | Suite No. 2 in B minor, BWV 1067, 7. Badinerie | Baroque | 124 |
| Joel Perri | El canto de mi Antara | Indio folk | 128 |
| Paul Desmond | Take Five | Jazz | 169 |
| ***Music-like noise* stimuli** | | | |
| Remo Giazotto | Adagio in G minor “Albinoni” | Neo-Baroque | 73 (by Shepard tones) |
| Ludwig van Beethoven | Symphony No. 7 in A major, Op 92, 2. Allegretto | Classical | 105 (by Shepard tones) |
| Ludwig van Beethoven | Piano Sonata No. 14 in C-sharp minor, Op. 27, No. 2 Allegretto | Classical | 117 (by Shepard tones) |
| Gustav Mahler | Symphony No. 5, 4. Adagietto | Romantic | 124 (by Shepard tones) |
| Duke Ellington | In a Sentimental Mood | Jazz | 128 (by Shepard tones) |
| Samuel Barber | Adagio for Strings | Neo-Romantic | 169 (by Shepard tones) |
