# Supplementary material for: Autonomic Effects of Music in Health and Crohn's Disease: The Impact of Isochronicity, Emotional Valence, and Tempo
Source: PLoS One. 2015 May 8;10(5):e0126224. doi: 10.1371/journal.pone.0126224 (PMC4425535; doi:10.1371/journal.pone.0126224)
Supplement: S2 Table — (DOCX) [file pone.0126224.s012.docx]

**S2 Table. Heart rate results of Experiment 1.**

| Analysis | Statistics | Significance |
| --- | --- | --- |
| **Comparison to silence (*Mdn* = 68, *IQR* = 12.7), planned ANOVA contrasts** | | |
| Pleasant music (*Mdn* = 70.5, *IQR* = 13.9) | *F*(1, 75) = 83.77 | *p* < .001, *r* = .73 |
| Isochronous tones (*Mdn* = 69, *IQR* = 14.5) | *F*(1, 75) = 116.06 | *p* < .001, *r* = .78 |
| Music-like noise (*Mdn* = 68.8, *IQR* = 14.7) | *F*(1, 75) = 83.61 | *p* < .001, *r* = .73 |
| **Post-hoc comparison between stimulus conditions, *p*-values Bonferroni-corrected if significant** | | |
| Pleasant music vs. Isochronous tones | *M_diff_* = -.006 [-.01,-.002] | *p* = .001 |
| Pleasant music vs. Music-like noise | *M_diff_* = -.006 [-.01,-.002] | *p* < .001 |
| Isochronous tones vs. Music-like noise | *M_diff_* = <.001 [-.003, .002] | *p* = .53 |

*Mdn*: median in min^-1^; *IQR*: interquartile range in min^-1^; *M_diff_*: mean difference of estimated marginal means of interbeat intervals in ms (lg_10_) [95% confidence interval of mean difference].

Effect size *r* > .5 indicates large effect.
