# Supplementary material for: Autonomic Effects of Music in Health and Crohn's Disease: The Impact of Isochronicity, Emotional Valence, and Tempo
Source: PLoS One. 2015 May 8;10(5):e0126224. doi: 10.1371/journal.pone.0126224 (PMC4425535; doi:10.1371/journal.pone.0126224)
Supplement: S3 Table — ANOVA main effects and planned comparisons. (DOCX) [file pone.0126224.s013.docx]

**S3 Table. Heart rate variability results of Experiment 1. ANOVA main effects and planned comparisons.**

| HRV parameter | Main effect | Sphericity correction | Pleasant music vs. silence | Isochronous tones vs. silence | Music-like noise vs. silence |
| --- | --- | --- | --- | --- | --- |
| SDNN***‡‡‡ | *F*(2.77, 207.72) = 48.55, *p* < .001 | Huynh-Feldt *ε* = .92 | *F*(1, 75) = 109.03, *p* < .001, *r* = .77 | *F*(1, 75) = 96.55, *p* < .001, *r* = .75 | *F*(1, 75) = 49.03, *p* < .001, *r* = .63 |
| RMSSD***‡‡‡ | *F*(2.51, 188.52) = 50.78, *p* < .001 | Huynh-Feldt *ε* = .84 | *F*(1, 75) = 101, *p* < .001, *r* = .76 | *F*(1, 75) = 76.11, *p* < .001, *r* = .71 | *F*(1, 75) = 41.14, *p* < .001, *r* = .6 |
| HF***‡‡‡ | *F*(2.78, 208.68) = 76.27, *p* < .001 | Huynh-Feldt *ε* = .93 | *F*(1, 75) = 175.31, *p* < .001, *r* = .84 | *F*(1, 75) = 98.05, *p* < .001, *r* = .75 | *F*(1, 75) = 61.23, *p* < .001, *r* = .67 |
| HF n.u.***‡‡‡ | *F*(2.53, 189.42) = 14.43, *p* < .001 | Huynh-Feldt *ε* = .84 | *F*(1, 75) = 24.93, *p* < .001, *r* = .5 | *F*(1, 75) = 2.68, *p* = .11 | *F*(1, 75) = 6.11, *p*= .016, *r* = .27 |
| LF***‡‡‡ | *F*(2.56, 192.05) = 35.24, *p* < .001 | Huynh-Feldt *ε* = .85 | *F*(1, 75) = 103.14, *p* < .001, *r* = .76 | *F*(1, 75) = 92.71, *p* < .001, *r* = .74 | *F*(1, 75) = 27.41, *p* < .001, *r* = .52 |
| LF n.u.***‡‡‡ | *F*(2.77, 207.6) = 13.08, *p* < .001 | Huynh-Feldt *ε* = .92 | *F*(1, 75) = 21.93, *p* < .001, *r* = .48 | *F*(1, 75) = 24.06, *p* < .001, *r* = .49 | *F*(1, 75) = 13.78, *p* < .001, *r* = .39 |
| LF/HF**‡ | *F*(2.54, 190.38) = 4.36, *p* = .008 | Huynh-Feldt *ε* = .85 | *F*(1, 75) = .38, *p* = .54 | *F*(1, 75) = 6.07, *p* = .016, *r* = .27 | *F*(1, 75) = .53, *p* = .47 |
| SD 1***‡‡‡ | *F*(2.52, 188.71) = 47.65, *p* < .001 | Huynh-Feldt *ε* = .84 | *F*(1, 75) = 92.44, *p* < .001, *r* = .74 | *F*(1, 75) = 78.86, *p* < .001, *r* = .72 | *F*(1, 75) = 39.11, *p* < .001, *r* = .59 |
| SD 2***‡‡‡ | *F*(2.79, 209) = 47.32, *p* < .001 | Huynh-Feldt *ε* = .93 | *F*(1, 75) = 105.99, *p* < .001, *r* = .77 | *F*(1, 75) = 95.1, *p* < .001, *r* = .75 | *F*(1, 75) = 52.25, *p* < .001, *r* = .64 |

**: *p* < .01; ***: *p*< .001 for main effect. ‡: *p < .*05; ‡‡‡: *p*< .001 for one or more contrasts.

Effect size *r* > .1 indicates small effect; *r* > .3 indicates medium effect; *r* > .5 indicates large effect.
