# Supplementary material for: Autonomic Effects of Music in Health and Crohn's Disease: The Impact of Isochronicity, Emotional Valence, and Tempo
Source: PLoS One. 2015 May 8;10(5):e0126224. doi: 10.1371/journal.pone.0126224 (PMC4425535; doi:10.1371/journal.pone.0126224)
Supplement: S4 Table — ANOVA post-hoc analyses, mean differences of estimated marginal means [95% confidence intervals of mean difference]. (DOCX) [file pone.0126224.s014.docx]

**S4 Table. Heart rate variability results of Experiment 1. ANOVA post-hoc analyses, mean differences of estimated marginal means [95% confidence intervals of mean difference].**

| HRV parameter | Pleasant music vs. Isochronous tones | Pleasant music vs. Music‑like noise | Music-like noise vs. Isochronous tones |
| --- | --- | --- | --- |
| SDNN* | -.02 [-.05, .01], *p* = .03 (corr. alpha level .008) | -.03 [-.06, -.003], *p* = .02 | .01 [-.01, .03], *p* = .17 |
| RMSSD*** | -.04 [-.06, -.01], *p* < .001 | -.04 [-.07, -.02], *p* < .001 | .01 [-.02, .03], *p* = .5 |
| HF*** | -.11 [-.15, -.07], *p* < .001 | -.12 [-.18, -.06], *p* < .001 | .01 [-.04, .06], *p* = .6 |
| HF n.u.*** | -.05 [-.08, -.02], *p* < .001 | -.04 [-.06, -.01], *p* <.001 | -.01 [-.04, .01], *p* = .18 |
| LF | -.04 [-.1, .02], *p* = .07 | -.08 [-.17, -.001], *p* = .008 (corr. alpha level .008) | .05, [-.03, .12], *p* = .13 |
| LF n.u. | .01 [-.01, .03], *p* = .54 | -.01 [-.03, .01], *p* = .31 | .01 [-.01, .04], *p* = .09 |
| LF/HF** | .06 [.02, .1], *p* = .001 | .03 [-.01, .06], *p* = .06 | .03 [-.01, .07], *p* = .03 (corr. alpha level .008) |
| SD 1*** | -.03 [-.05, -.01], *p* = .002 | -.04 [-.06, -.01], *p* <. 001 | .01 [-.01, .03], *p* = .32 |
| SD 2* | -.02 [-.05, -.01], *p* = .06 | -.03 [.06, -.001], *p* = .047 | .01 [-.01, .03], *p* = .19 |

*: *p < .*05; **: *p* < .01; ***: *p* < .001 for one or more pairwise comparison; *p*-values Bonferroni-corrected unless not significant (to avoid misinterpretation regarding beta-error estimation).
