# Supplementary material for: Autonomic Effects of Music in Health and Crohn's Disease: The Impact of Isochronicity, Emotional Valence, and Tempo
Source: PLoS One. 2015 May 8;10(5):e0126224. doi: 10.1371/journal.pone.0126224 (PMC4425535; doi:10.1371/journal.pone.0126224)
Supplement: S5 Table — (DOCX) [file pone.0126224.s015.docx]

**S5 Table. Electrodermal activity results of Experiment 1.**

| Analysis | Statistic | Significance | Effect size |
| --- | --- | --- | --- |
| **Comparison to silence (*Mdn* = .38, *IQR* = .9), Wilcoxon signed-rank tests, *p*-values Bonferroni-corrected** | | | |
| Pleasant music (*Mdn* = 1.26, *IQR* = 2.43) | *z* = -5.74 | *p* < .001 | *r* = .48 |
| Isochronous tones (*Mdn* = .56, *IQR* = .97) | *z* = -2.49 | *p* = .049 | *r* = .21 |
| Music-like noise (*Mdn* = .65, *IQR* = 1.08) | *z* = -3.82 | *p* < .001 | *r* = .32 |
| **Comparison between pleasant and unpleasant, Wilcoxon signed-rank tests, *p*-value Bonferroni-corrected** | | | |
| Pleasant music vs. Music-like noise | *z* = -5.12 | *p* < .001 | *r* = .43 |

*Mdn*: median of sum of EDA amplitudes; *IQR*: interquartile range of sum of EDA amplitudes.

Effect size *r* > .1 indicates small effect; *r* > .3 indicates medium effect.
