# Supplementary material for: Autonomic Effects of Music in Health and Crohn's Disease: The Impact of Isochronicity, Emotional Valence, and Tempo
Source: PLoS One. 2015 May 8;10(5):e0126224. doi: 10.1371/journal.pone.0126224 (PMC4425535; doi:10.1371/journal.pone.0126224)
Supplement: S6 Table — (DOCX) [file pone.0126224.s016.docx]

**S6 Table. Pleasant music stimuli of Experiment 2.**

| Composer | Title | Style |
| --- | --- | --- |
| Georg Friedrich Händel | Recorder Sonata F major, HWV369, 2. Allegro | Baroque |
| Johann Sebastian Bach | Prelude C minor, BWV 847 | Baroque |
| Johann Sebastian Bach | Prelude C major, BWV 870 | Baroque |
| Johann Sebastian Bach | Fugue C major, BWV 953 | Baroque |
| Johann Sebastian Bach | Fugue G minor, BWV 861 | Baroque |
| Johann Sebastian Bach | Fugue A minor, BWV 865 | Baroque |
| Karl Teike | Graf Zeppelin (The Conqueror) | March music |
| James H. Marshall, Walter Wolff | Evening Pastimes | Ragtime |
| Scott Joplin | Eugenia | Ragtime |
| Scott Joplin | The Easy Winners | Ragtime |
