# Supplementary material for: Autonomic Effects of Music in Health and Crohn's Disease: The Impact of Isochronicity, Emotional Valence, and Tempo
Source: PLoS One. 2015 May 8;10(5):e0126224. doi: 10.1371/journal.pone.0126224 (PMC4425535; doi:10.1371/journal.pone.0126224)
Supplement: S7 Table — (DOCX) [file pone.0126224.s017.docx]

**S7 Table. Heart rate results of Experiment 2.**

| Analysis | Statistics | Significance |
| --- | --- | --- |
| **Comparison to silence (*M* = 61.5, *SD* = 8.4), planned ANOVA contrasts** | | |
| Slow pleasant music (*M* = 63, *SD* = 9.3) | *F*(1, 28) = 3.99 | *p* = .06 |
| Fast pleasant music (*M* = 62.2, *SD* = 7.9 | *F*(1, 28) = .7 | *p* = .41 |
| Slow unpleasant music (*M* = 63.5, *SD* = 9.2) | *F*(1, 28) = 5.54 | *p* = .03, *r* = .41 |
| Fast unpleasant music(*M* = 62.1, *SD* = 8) | *F*(1, 28) = .38 | *p* = .54 |
| **Post-hoc comparison between tempi, *p*-values not Bonferroni-corrected** | | |
| Slow pleasant music vs. Fast pleasant music | *M_diff_* = -9.34 [-58.56, 39.87] | *p* = .57 |
| Slow unpleasant music vs. Fast unpleasant music | *M_diff_* = 19.47 [-73.38, 34.45] | *p* = .28 |
| **Post-hoc comparison between pleasant and unpleasant, *p*-values not Bonferroni-corrected** | | |
| Slow pleasant music vs. Slow unpleasant music | *M_diff_* = 8.41 [-11.04, 27.85] | *p* = .2 |
| Fast pleasant music vs. Fast unpleasant music | *M_diff_* = -1.72 [-11.56, 8.12] | *p* = .6 |

*M*: mean in min^-1^; *SD*: standard deviation in min^-1^; *M_diff_*: mean difference of estimated marginal means of interbeat intervals in ms [95% confidence interval of mean difference].

Effect size *r* > .3 indicates medium effect.
