# Supplementary material for: Autonomic Effects of Music in Health and Crohn's Disease: The Impact of Isochronicity, Emotional Valence, and Tempo
Source: PLoS One. 2015 May 8;10(5):e0126224. doi: 10.1371/journal.pone.0126224 (PMC4425535; doi:10.1371/journal.pone.0126224)
Supplement: S8 Table — ANOVA main effects and planned comparisons. (DOCX) [file pone.0126224.s018.docx]

**S8 Table. Heart rate variability results of Experiment 2. ANOVA main effects and planned comparisons.**

| HRV parameter | Main effect | Sphericity correction | Fast pleasant music vs. silence | Slow pleasant music vs. silence | Fast unpleasant music vs. silence | Slow unpleasant music vs. silence |
| --- | --- | --- | --- | --- | --- | --- |
| SDNN**‡‡‡ | *F*(2.57, 71.91) = 6.94, *p* = .001 | Greenhouse-Geisser *ε* = .64 | *F*(1, 28) = 12.45, *p* = .001, *r* = .55 | *F*(1, 28) = 17.81, *p* < .001, *r* = .62 | *F*(1, 28) = 12.62, *p* = .001, *r* = .56 | *F*(1, 28) = 5.9, *p*= .02, *r* = .42 |
| RMSSD | *F*(2.29, 64.19) = .93, *p* = .41 | Greenhouse-Geisser *ε* = .57 | *F*(1, 28) = .89, *p* = .35 | *F*(1, 28) = 1.62, *p* = .21 | *F*(1, 28) = 1.47, *p* = .24 | *F*(1, 28) = .01, *p* = .91 |
| HF*‡ | *F*(2.3, 64.37) = 3.32, *p* = .036 | Greenhouse-Geisser *ε* = .58 | *F*(1, 28) = 5.16, *p* = .031, *r* = .39 | *F*(1, 28) = 6.14, *p* = .019, *r* = .42 | *F*(1, 28) = 5.78, *p* = .023, *r* = .41 | *F*(1, 28) = 2.96, *p* = .1 |
| HF n.u. | *F*(2.51, 70.34) = .28, *p* = .8 | Greenhouse-Geisser *ε* = .63 | *F*(1, 28) = .05, *p* = .82 | *F*(1, 28) = .12, *p*= .73 | *F*(1, 28) = .004, *p* = .95 | *F*(1, 28) = .86, *p* = .36 |
| LF***‡‡‡ | *F*(1.89, 52.91) = 11.05, *p* < .001 | Greenhouse-Geisser *ε* = .47 | *F*(1, 28) = 16.64, *p* < .001, *r* = .61 | *F*(1, 28) = 13.43, *p* = .001, *r* = .57 | *F*(1, 28) = 16.75, *p* < .001, *r* = .61 | *F*(1, 28) = 12.52, *p* = .001, *r* = .56 |
| LF n.u.***‡‡‡ | *F*(2.42, 67.61) = 34.54, *p* < .001 | Greenhouse-Geisser *ε* = .6 | *F*(1, 28) = 59.81, *p* < .001, *r* = .83 | *F*(1, 28) = 76.58, *p* < .001, *r* = .86 | *F*(1, 28) = 51.39, *p* < .001, *r* = .8 | *F*(1, 28) = 39.75, *p* < .001. *r* = .77 |
| LF/HF***‡‡‡ | *F*(2.47, 69.26) = 14.5, *p* < .001 | Greenhouse-Geisser *ε* = .62 | *F*(1, 28) = 24.81, *p* < .001, *r* = .69 | *F*(1, 28) = 22.24, *p* < .001, *r* = .67 | *F*(1, 28) = 26.09, *p* < .001, *r* = .69 | *F*(1, 28) = 20.62, *p* < .001, *r* = .65 |
| SD 1 | *F*(2.28, 63.82) = 1.05, *p* = .36 | Greenhouse-Geisser *ε* = .57 | *F*(1, 28) = 1.26, *p* = .27 | *F*(1, 28) = 2.23, *p* = .15 | *F*(1, 28) = 1.82, *p* = .19 | *F*(1, 28) = .12, *p* = .74 |
| SD 2***‡‡‡ | *F*(2.58, 72.37) = 20.41, *p* < .001 | Greenhouse-Geisser *ε* = .65 | *F*(1, 28) = 33.49, *p* < .001, *r* = .74 | *F*(1, 28) = 33.85, *p* < .001, *r* = .74 | *F*(1, 28) = 35.67, *p* < .001, *r* = .75 | *F*(1, 28) = 24.23, *p* < .001, *r* = .68 |

*: *p <*.05; **: *p* < .01; ***: *p* < .001 for main effect; ‡: *p* < .05; ‡‡: *p* < .01; ‡‡‡: *p*< .001 for one or more contrasts.

Effect size *r* > .3 indicates medium effect; *r* > .5 indicates large effect.
