# Supplementary material for: Autonomic Effects of Music in Health and Crohn's Disease: The Impact of Isochronicity, Emotional Valence, and Tempo
Source: PLoS One. 2015 May 8;10(5):e0126224. doi: 10.1371/journal.pone.0126224 (PMC4425535; doi:10.1371/journal.pone.0126224)
Supplement: S9 Table — ANOVA post-hoc analyses, mean differences of estimated marginal means [95% confidence intervals of mean difference], p-values Bonferroni-corrected. (DOCX) [file pone.0126224.s019.docx]

**S9 Table. Heart rate variability results of Experiment 2. ANOVA post-hoc analyses, mean differences of estimated marginal means [95% confidence intervals of mean difference].**

| HRV parameter | Slow pleasant music vs. Fast pleasant music | Slow unpleasant music vs. Fast unpleasant music | Slow pleasant music vs. Slow unpleasant music | Fast pleasant music vs. Fast unpleasant music |
| --- | --- | --- | --- | --- |
| SDNN | .001 [-.07, .07], *p* = .96 | .04 [-.05, .13], *p* = .19 | -.03 [-.1, .04], *p* = .2 | .01 [-.03, .05], *p* = .49 |
| RMSSD | .002 [-.08, .08], *p* = .95 | .05 [-.06, .16], *p* = .16 | -.03 [-.11, .05], *p* = .24 | .02 [-.04, .07], *p* = .34 |
| HF | .01 [-.08, .09], *p* = .83 | .03 [-.07, .13], *p* = .41 | -.02 [-.12, .09], *p* = .63 | .01 [-.08, .09], *p* = .87 |
| HF n.u. | .002 [-.07, .08], *p* = .95 | .02 [-.05, .09], *p* = .36 | -.01 [-.05, .03], *p* = .37 | .01 [-.03, .05], *p* = .57 |
| LF | .01 [-.1, .12], *p* = .73 | .01 [-.09, .1], *p* = .83 | -.01 [-.12, .09], *p* = .7 | -.02 [-.12, .08], *p* = .56 |
| LF n.u. | .004 [-.03, .03], *p* = .73 | .01 [-.02, .04], *p* = .31 | -.01 [-.04, .02], *p* = .52 | <.001 [-.02, .02], *p* = .97 |
| LF/HF | .01 [-.07, .09], *p* = .76 | -.004 [-.08, .07], *p* = .87 | .01 [-.04, .05], *p* = .68 | -.01 [-.06, .04], *p* = .73 |
| SD 1 | .001 [-.08, .08], *p* = .99 | .05 [-.06, .15], *p* = .19 | -.03 [-.11, .05], *p* = .26 | .02 [-.04, .07], *p* = .36 |
| SD 2 | -.001 [-.06, .06], *p* = .97 | .03 [-.03, .08], *p* = .15 | -.02 [-.07, .03], *p* = .2 | .01 [-.03, .04], *p* = .63 |

Not significant *p*-values not Bonferroni-corrected to avoid misinterpretation regarding beta-error estimation.
