# Supplementary material for: Autonomic Effects of Music in Health and Crohn's Disease: The Impact of Isochronicity, Emotional Valence, and Tempo
Source: PLoS One. 2015 May 8;10(5):e0126224. doi: 10.1371/journal.pone.0126224 (PMC4425535; doi:10.1371/journal.pone.0126224)
Supplement: S10 Table — ANOVA main effects, planned contrasts, group effects, and interaction effects. (DOCX) [file pone.0126224.s020.docx]

**S10 Table. Heart rate variability results of Experiment 3. ANOVA main effects, planned contrasts, group effects, and interaction effects.**

| HRV parameter | Main effect | Sphericity correction | Pleasant music vs silence | Music-like noise vs silence | Group effect | Interaction effect |
| --- | --- | --- | --- | --- | --- | --- |
| SDNN***‡‡‡ | *F*(1.81, 88.9) = 11.86, *p* < .001 | Huynh-Feldt *ε*= .91 | *F*(1, 49) = 14.43, *p* < .001, *r* = .48 | *F*(1, 49) = 15.16, *p* < .001, *r* = .49 | *F*(1, 49) = .002, *p* = .96 | *F*(1.81, 88.9) = .05, *p* = .94 |
| RMSSD**‡‡‡ | *F*(1.36, 66.38) = 10.46, *p* = .001 | Greenhouse-Geisser *ε* = .68 | *F*(1, 49) = 14.98, *p* < .001, *r* = .48 | *F*(1, 49) = 9.02, *p* = .004, *r* = .39 | *F*(1, 49) = .54, *p*= .47 | *F*(1.36, 66.38) = .48, *p* = .55 |
| HF***‡‡‡ | *F*(1.83, 89.88) = 12.86, *p* < .001 | Huynh-Feldt *ε*= .92 | *F*(1 ,49) = 25.98, *p* < .001, *r* = .59 | *F*(1, 49) = 9.74, *p* = .003, *r* = .41 | *F*(1, 49) = .29, *p*= .59 | *F*(1.83, 89.88) = 1.34, *p* = .26 |
| HFnu**‡ | *F*(2, 98) = 4.91, *p* = .009 | Mauchly´s *p* = .07 | *F*(1, 49) = 7.16, *p* = .01, *r* = .36 | *F*(1, 49) = .41, *p* = .53 | *F*(1, 49) = 2.62, *p* = .11 | *F*(2, 98) = .37, *p*= .69 |
| LF***‡‡‡ | *F*(1.85, 90.38) = 10.85, *p* < .001 | Huynh-Feldt *ε* = .92 | *F*(1, 49) = 16.5, *p* < .001, *r* = .5 | *F*(1 ,49) = 12.08, *p* = .001, *r* = .44 | *F*(1, 49) = .11, *p*= .75 | *F*(1.85, 90.38) = .47, *p* = .61 |
| LFnu*‡ | *F*(2, 98) = 4.56, *p* = .013 | Mauchly´s *p* = .3 | *F*(1, 49) = 6.1, *p*= .017, *r* = .33 | *F*(1, 49) = 6.52, *p* = .014, *r* = .34 | *F*(1, 49) = 2.48, *p* = .12 | *F*(2, 98) = .84, *p* = .44 |
| LF/HF | *F*(2, 98) = 1.37, *p* = .26 | Mauchly´s *p* = .15 | *F*(1, 49) = .003, *p* = .96 | *F*(1 ,49) = 1.75, *p* = .19 | *F*(1, 49) = 3.88, *p* = .06 | *F*(2, 98) = .38, *p* = .69 |
| SD1**‡‡ | *F*(1.36, 66.56) = 8.78, *p* = .002 | Greenhouse-Geisser *ε* = .68 | *F*(1, 49) = 12.42, *p* = .001, *r* = .43 | *F*(1 ,49) = 8.03, *p* = .007, *r* = .38 | *F*(1, 49) = .52, *p*= .48 | *F*(1.36, 66.56) = .43, *p* = .58 |
| SD2***‡‡‡ | *F*(2, 98) = 11.96, *p* < .001 | Mauchly´s *p* = .08 | *F*(1, 49) = 14.31, *p* < .001, *r* = .48 | *F*(1, 49) = 16.38, *p* < .001, *r* = .5 | *F*(1, 49) = .08, *p*= .78 | *F*(2, 98) = .02, *p*= .98 |

*: *p < .*05; **: *p* < .01; ***: *p*< .001 for main effect; ‡: *p* < .05; ‡‡: *p* < .01; ‡‡‡: *p*< .001 for one or more contrasts.

Effect size *r* > .3 indicates medium effect; *r* > .5 indicates large effect.
