# Supplementary material for: Autonomic Effects of Music in Health and Crohn's Disease: The Impact of Isochronicity, Emotional Valence, and Tempo
Source: PLoS One. 2015 May 8;10(5):e0126224. doi: 10.1371/journal.pone.0126224 (PMC4425535; doi:10.1371/journal.pone.0126224)
Supplement: S11 Table — ANOVA post-hoc analyses, mean differences of estimated marginal means [95% confidence intervals of mean difference]. (DOCX) [file pone.0126224.s021.docx]

**S11 Table. Heart rate variability results of Experiment 3. ANOVA post-hoc analyses, mean differences of estimated marginal means [95% confidence intervals of mean difference].**

| HRV parameter | Pleasant music vs. Music‑like noise |
| --- | --- |
| SDNN | .01 [-.02, .04], *p* = .55 |
| RMSSD | -.01 [-.03, .01], *p* = .36 |
| HF | -.04 [-.1, .03], *p* = .16 |
| HF n.u.* | -.03 [-.06, .004], *p* = .02 |
| LF | -.002 [-.08, .07], *p* = .95 |
| LF n.u. | -.003 [-.03, .02], *p* = .81 |
| LF/HF | .03 [-.01, .07], *p* = .09 |
| SD°1 | -.004 [-.03, .02], *p* = .65 |
| SD°2 | .01 [-.03, .05], *p* = .45 |

*: *p* < .05; not significant *p*-values not Bonferroni-corrected to avoid misinterpretation regarding beta-error estimation.
